# On electromagnetic head digitization in MEG and EEG

**DOI:** 10.1038/s41598-023-30223-9

**Published:** 2023-03-07

**Authors:** Amit Jaiswal, Jukka Nenonen, Lauri Parkkonen

**Affiliations:** 1 MEGIN Oy, Espoo, Finland; 2 Department of Neuroscience and Biomedical Engineering, School of Science, Aalto University, Espoo, Finland

**Keywords:** Applied physics, Techniques and instrumentation, Neuroscience, 3-D reconstruction, Magnetoencephalography, Electroencephalography - EEG, Biomedical engineering

## Abstract

In MEG and EEG studies, the accuracy of the head digitization impacts the co-registration between functional and structural data. The co-registration is one of the major factors that affect the spatial accuracy in MEG/EEG source imaging. Precisely digitized head-surface (scalp) points do not only improve the co-registration but can also deform a template MRI. Such an individualized-template MRI can be used for conductivity modeling in MEG/EEG source imaging if the individual’s structural MRI is unavailable. Electromagnetic tracking (EMT) systems (particularly Fastrak, Polhemus Inc., Colchester, VT, USA) have been the most common solution for digitization in MEG and EEG. However, they may occasionally suffer from ambient electromagnetic interference which makes it challenging to achieve (sub-)millimeter digitization accuracy. The current study—(i) evaluated the performance of the Fastrak EMT system under different conditions in MEG/EEG digitization, and (ii) explores the usability of two alternative EMT systems (Aurora, NDI, Waterloo, ON, Canada; Fastrak with a short-range transmitter) for digitization. Tracking fluctuation, digitization accuracy, and robustness of the systems were evaluated in several test cases using test frames and human head models. The performance of the two alternative systems was compared against the Fastrak system. The results showed that the Fastrak system is accurate and robust for MEG/EEG digitization if the recommended operating conditions are met. The Fastrak with the short-range transmitter shows comparatively higher digitization error if digitization is not carried out very close to the transmitter. The study also evinces that the Aurora system can be used for MEG/EEG digitization within a constrained range; however, some modifications would be required to make the system a practical and easy-to-use digitizer. Its real-time error estimation feature can potentially improve digitization accuracy.

## Introduction

In electromagnetic functional source imaging such as magnetoencephalography (MEG) and electroencephalography (EEG), anatomical MRI (magnetic resonance imaging) images from an individual’s head are usually utilized for defining the geometry and head model for the computations. A reliable fusion of the functional and anatomical information requires that the locations of anatomical landmarks are known accurately on the head surface. Also, the locations and orientations of the sensors/electrodes with respect to the head need to be known with sufficient accuracy^1^. Since the functional and anatomical data are acquired by two separate medical imaging systems, their combination requires *co-registering* their coordinate frames to define an affine transformation between MEG/EEG and MRI coordinate systems. This co-registration is usually done by manually aligning a set of fiducial points determined in the two coordinate frames. Three easily identifiable anatomical landmarks on MRI and the scalp surface—nasion, left preauricular (LPA), and right preauricular (RPA)—typically serve as the fiducial points for the co-registration. Optionally, using an ICP-based (iterative closest point)^2^ automated co-registration, a set of scalp surface points determined in the MEG/EEG coordinate frame can be matched with the scalp surface extracted from MRI.

**Figure 1:**
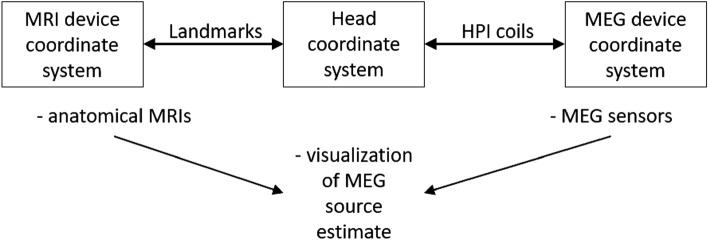
The coordinate systems and their relations^3^.

The fiducial points in the *MRI coordinate frame* are determined by visually navigating the 3D MRI images or the scalp surface extracted from those images. In the MEG/EEG coordinate frame, these fiducial positions and the scalp surface points are determined using an electromagnetic or optical position tracking device during a procedure generally referred to as *head digitization*. Most MEG systems are based on a fixed sensor array with precisely defined sensor positions in the *MEG device coordinate frame*, and the head can potentially move wrt. the sensors during the data acquisition. In the MEG systems by MEGIN Oy (Espoo, Finland), the location of the subject’s head relative to the MEG sensors is determined with the aid of four or five head position indicator (HPI) coils attached to the scalp. The HPI coil locations are digitized along with the fiducial and scalp points, and their positions are determined in the *head coordinate frame* defined by the digitized fiducial points. When the coils are energized, the MEG sensor array can localize them in the MEG device coordinate frame. Knowing the HPI coil locations in these two frames, a device-to-head coordinate transformation is defined that determines the head position inside the MEG sensor array. Thus, MEG source imaging studies involve a combination of three coordinate systems (Fig. 1)^3^:

i. MEG device coordinate system that defines the MEG sensor locations and orientations with respect to each other.
ii. Head coordinate system based on the digitized fiducial points identifiable accurately on the head surface and in the MRI data.
iii. MRI coordinate system, which can be native (device-based) or determined by MRI processing software.

EEG electrodes are digitized in the same head coordinate frame along with the other digitized points because they are on the scalp. Thus, EEG source imaging involves a combination of only two coordinate systems—the head coordinate system and the MRI coordinate system.

Earlier studies have investigated the effect of co-registration accuracy in the MEG/EEG source imaging^4–6^ and they confirm that careful and accurate digitization is a crucial step. In MEG/EEG, digitization is primarily used for defining the coordinate transformations, either during data acquisition or source estimation, and sometimes to fit a spherical head model for source modeling^7,8^. However, some studies have also demonstrated the role of digitized points in defining a pseudo-realistic volume conductor model^9–11^. A realistic head model using an individual’s inner skull and scalp surfaces is shown to outperform the spherical head model in source imaging^12,13^. However, defining a realistic volume conductor model is sometimes challenging, especially when an individual’s MRI is unavailable. In such a case, e.g., the TPS (thin-plate spline)^14^ method can be applied to warp template scalp and brain surfaces using densely sampled scalp digitization points from the subject’s head. Although the individualized (warped) surfaces would not be precisely the real individual MRI, they are sufficiently similar to be useful in MEG/EEG analysis. Since digitization is influential in both the coordinate transformation and the warping approach, digitization accuracy is crucial in MEG/EEG source imaging.

Various techniques have been introduced for scalp digitization and localization of EEG electrodes. In addition, a few studies have evaluated the advantages and limitations of different digitization methods^15,16^. These methods include:

- Digital calipers to localize electrodes by measuring distances between electrode pairs and fiducial points^17,18^.
- MRI marker capsules attached to electrodes^19,20^.
- Ultrasonic digitization^15,21^.
- Photogrammetry-based digitization^22–24^.
- Electromagnetic tracking^25,26^.
- Laser-based optical scanning^27,28^.
- Reflective marker-based optical scanning^16^.
- Structured-light 3D scanning^29–31^.

Among these methods, EMT-based digitization has been the most common approach for a long time. An EMT is a transmitter–receiver -based tracking system, where a receiver is placed in an electromagnetic (EM) field generated by the transmitter for determining the receiver’s three-dimensional position with respect to the transmitter or another receiver. However, a nearby magnetic object may distort the transmitter field and deteriorate digitization accuracy. Also, EMT takes longer for dense EEG electrode setups, as the receiver must be pointed to each electrode. As an alternative to EMT, optical scanners have been introduced in recent years. They can make the digitization process faster and are free of EM interference issues, but generally suffer from the *line-of-sight* problem. IR-reflector-based scanners such as Krios® (NDI, Waterloo, ON, Canada) have been developed as alternate solutions in recent years, especially for a dense EEG cap^16^, they offer millimeter-level accuracy and faster digitization for dense EEG. However, during MEG scans, an EEG cap is often not worn; as a result, the optical scanner only operates in a probing mode, similar to an EMT-based digitizer, and the line-of-sight issue worsens, making the digitization of scalp points more challenging and time-consuming. Recent studies have introduced the structured-light 3D scanner (Occipital, CO, USA) for digitization^29–31^. Such a scanner is fast and provides a dense triangular mesh of the head surface with high accuracy, particularly at smoothly varying locations, but post-processing is required to identify the anatomical landmarks. Furthermore, as the scanner yields an envelope of hair instead of scalp, a different co-registration strategy utilizing solely the non-hairy head surface is needed. Additionally, since the scalp may not be accurately defined due to hair, correct warping of a template MRI is impossible.

Due to the above practical constraints of optical digitizers, EMT systems such as Fastrak® or its older variants (Polhemus Inc., Colchester, VT, USA) are still the most widely used digitizers in MEG/EEG and may remain the most practical solution for MEG. The Fastrak® with the standard TX2 transmitter achieves millimeter precision and is widely accepted for digitization in MEG/EEG research and clinical practice^15,32^. However, some studies have reported variations in digitization accuracy up to several millimeters depending on measurement conditions^15,32^. Nearby EM interference sources, physical contact between transmitter and receiver cables, skin and hair softness, and head movements are the reported reasons behind such variations^32^. In a clinical environment, there can be additional factors distorting the transmitter EM field, such as dental work, long-term EEG monitoring electrodes, magnetized material from prior surgery, and nearby immovable instruments. Surgical implants and therapeutic stimulators are unavoidable sources of artifacts. The magnetic properties of their materials and circuits may distort the transmitter field, lowering the digitization accuracy. The users may identify and rectify some of the interferences but conditions such as reinforcing metal bars in nearby walls or ceilings may reduce digitization precision covertly. Other practical limitations, such as hand tremors while digitizing fiducial points and overlapping transmitter–receiver cables, can also add some inaccuracy.

Non-systematic and site-specific variations in digitization accuracy have made it difficult to investigate the sources of interference, quantify their effects, and define a robust MEG/EEG digitization method. Also, it is crucial to evaluate the current system's performance in various interference cases and investigate options for developing a more robust system for digitization. The present study focuses on two aspects:

i. An extensive evaluation of the current EMT-based digitizer, Fastrak® with the TX2 transmitter, in various interference conditions, to determine best digitization practices during MEG/EEG.
ii. A side-by-side comparison of two different EMT-based systems to assess if there is a more robust digitizer for MEG/EEG.

For each of the three systems, the position tracking fluctuation^33,34^ was quantified both with and without the presence of different electromagnetic interferences. Further, the effects of different measurement conditions were extensively examined, such as varying transmitter–receiver distance, movement during digitization, nearby interference, surrounding infrastructural interference, and a medical implant. Two rigid test models with precisely defined points were used to test the digitization accuracy across several ambient conditions. We also tested the performance of the three digitization systems with a pre-marked human head model to replicate the free-form scalp digitization in MEG and a 32-channel EEG cap to test the fixed-electrode digitization in EEG. Finally, we showed the impact of the three ways of digitization on MEG source modeling. In addition to summarizing our findings, we provided some guidelines to follow when utilizing the EMT-based system for MEG/EEG digitization.

## Materials and methods

### EMT systems

The three EMT systems evaluated in the study were—(i) a Fastrak^®^ system with a standard (TX2) transmitter, hereafter referred to as *Fastrak TX2,* (ii) an Aurora^®^ system with a 20–20 planar field generator, hereafter referred to as *Aurora,* and (iii) a Fastrak® system with a short-range (TX1) transmitter, hereafter referred to as *Fastrak TX1*. Fastrak TX1 differed from Fastrak TX2 by using a short-range transmitter that is optimized for digitizing within ~ 50 cm and was expected to be less susceptible to EM interference sources beyond this range. The Aurora system has been used for several years in clinical applications where submillimeter spatial precision is required, e.g., surgical navigation; however, it has not been applied for MEG/EEG digitization. The hardware specifications, constrained EM field, expected accuracy, and a real-time error-indicating feature motivated us to investigate the Aurora system against the current digitizer for a potential new digitization option. Table 1 compares the main features of the three systems; a comparative illustration of their EM field is shown in Supplementary Fig. S1A. These three systems cover a wide range of applications, but in this study, we investigated them only as a digitizer for MEG/EEG, especially in MEG acquisition. Note that the transmitter is referred to as a field generator in Aurora system manuals (NDI, Waterloo, ON, Canada). The receiver as a tool and the stylus as a probe match the common terminology used for Fastrak TX2, and we used these terms for all three systems.

**Table 1:** Specifications of the systems used in the study.

	Fastrak TX2	Fastrak TX1	Aurora
Manufacturer	Polhemus Inc., Colchester, VT, USA	Polhemus Inc., Colchester, VT, USA	NDI, Waterloo, ON, Canada
System version	3.0	3.0	3.1
Main processing unit	SEU: system electronics unit	SEU: system electronics unit	SCU: system control unit, SIU: sensor interface unit
Transmitter	TX2 (standard)	TX1 (short-ranger)	20–20 PFG
Stylus length (cm)	18 (with a switch)	18 (with a switch)	20 (without a switch)
Transmitter dimensions (*l* × *b* × *h; *cm^3^)	5.8 × 5.1 × 5.8	2.3 × 2.8 × 1.5	20 × 20 × 7.1
Radius (cm) and shape of the Volume of measurement (VoM)	~ 150 spherical	~ 50 spherical	~ 66 dome-shaped, or ~ 55 cube-shaped
Operating frequency (Hz)	13,000	13,000	800
DOF (degree of freedom) of the receivers used	6	6	6
Sampling frequency (Hz)	120/no. of receiver	120/no. of receiver	40 per receiver
Interface with host computer	USB, RS232	USB, RS422	USB, RS422
Expected accuracy within VoM (mm)	< 1	< 1	< 1

### Fastrak TX2 system

**Figure 2:**
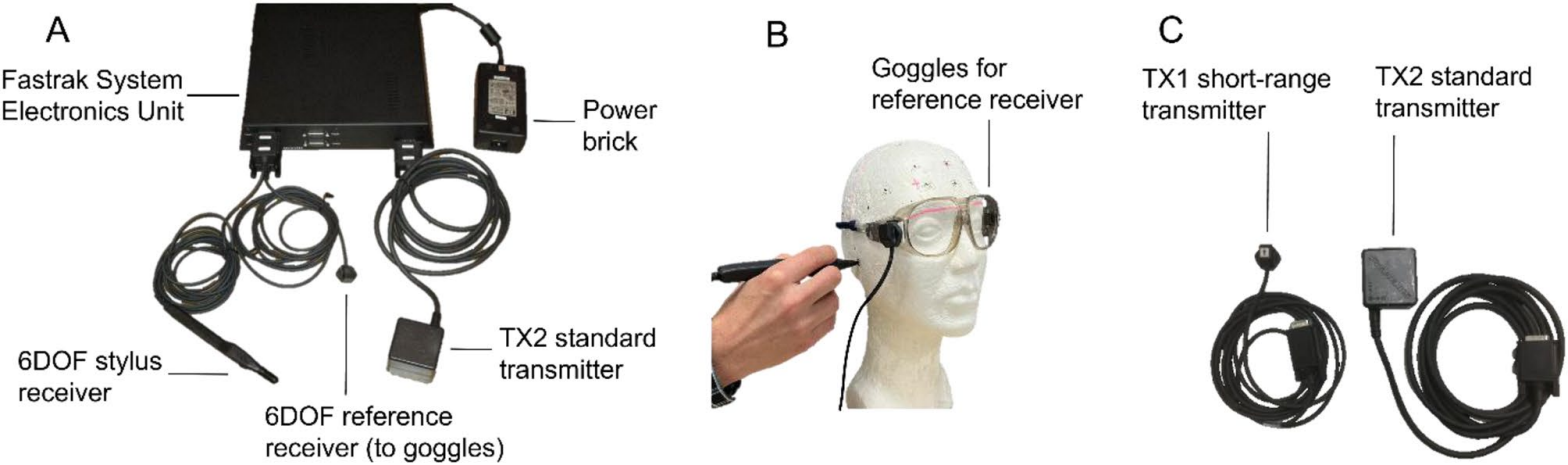
Fastrak system. (**A**) The standard TX2 system components, (**B**) reference receiver attached to googles during digitization, (**C**) physical differences between the TX1 and TX2 transmitters.

Fastrak TX2 is a general-purpose EMT system designed for various spatial tracking applications including MEG/EEG digitization. The standard transmitter TX2 generates a spherical field with substantial strength up to ~ 150 cm, and a much weaker field even longer, allowing a large VoM (volume of measurement) and more freedom for digitization around the head (3space^®^ Fastrak^®^ user's manual, Polhemus Inc., Colchester, VT, USA). Using AC electromagnetics, the processing unit SEU continuously tracks a *probe receiver* wrt. to a *reference receiver* at a maximum rate of 60 Hz. It provides an easy-to-use digitization platform using a switch-enabled probing receiver and MEG/EEG system-specific digitization interfaces. Fastrak TX2 is considered the gold standard for MEG/EEG digitization; therefore, we employed this system as a reference digitizer in the study. We used a TX2 transmitter and two receivers, i.e., a stylus and a reference receiver. The stylus is approximately an 18-cm long pen-like ‘probing tool’ with a switch expanding the system capability into a free-form digitizer where both single and continuous position output can be obtained. In the MEG/EEG application, the reference receiver is usually fixed with goggles (or an elastic band) to the patient's head so that it moves with the head. SEU calculates the receivers' position in the global (wrt. center of the transmitter) and referenced (wrt. center of the reference receiver) coordinate system. The digitization is done in the reference coordinate frame, centered at the reference receiver, and moving with the head to compensate for the head movement during digitization. The system-specific digitization software, for example, MEGIN’s *Isotrak server*, converts each digitized point into the head coordinate frame in real-time, just after the three fiducial points get digitized. Figure 2A shows the Fastrak TX2 setup used in the study, and Fig. 2B depicts the reference receiver attachment to the head.

### Fastrak TX1 system

Eddy currents in conducting objects play a major role in EMT field distortion^35^. They are proportionate to factors such as field strength, rate of change, object conductivity, and surface area. Therefore, the stronger EM field at the same distance from the transmitter TX2 compared to TX1 produces stronger eddy currents and so higher distortion. In practice, MEG/EEG digitization may not require such a large VoM that the TX2 provides. Instead, a transmitter with a smaller VoM with a radius below 100 cm might be optimal to cover the whole head and at the same time reduce the susceptibility to interference sources such as conducting materials in floor, walls, and recessed ceilings. Fastrak TX1 possesses a spherical field decaying over ~ 50 cm, making the system less susceptible to interference sources outside this range. Figure 2C shows the physical difference between the TX1 and TX2 transmitters. The rest of the setup and functioning were identical to the Fastrak TX2 system detailed in the previous section.

### Aurora system

**Figure 3:**
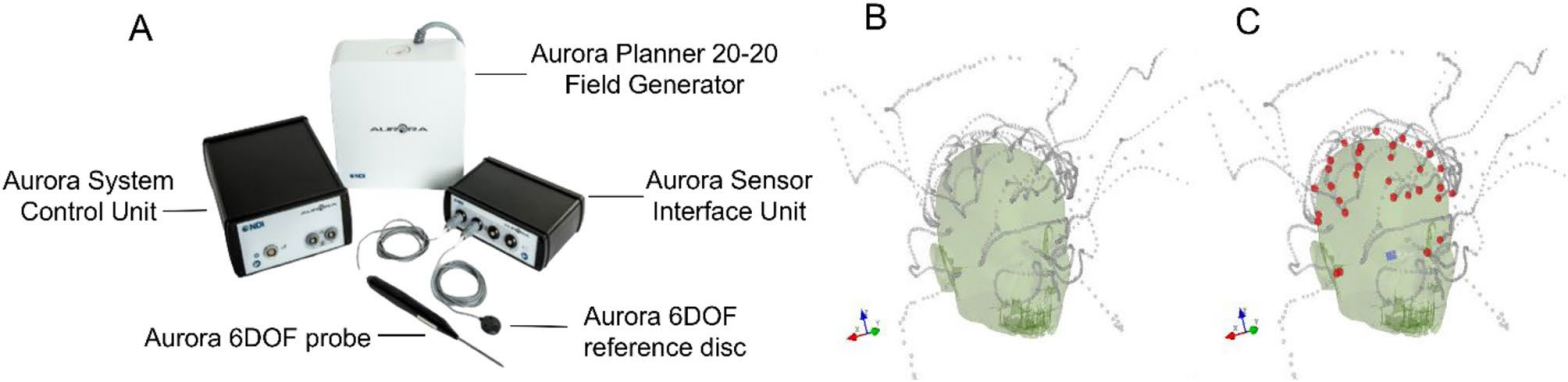
Aurora system. (**A**) Components used for the study (image courtesy to NDI, Waterloo, ON, Canada), (**B**) clustered continuous tracking points (grey points). (**C**) The digitization points (red); both in MEGIN’s head coordinate system.

Aurora is a general-purpose EMT system that has been utilized for many spatial tracking tasks including clinical applications requiring high spatial accuracy^33,36,37^. The system components used in this study are shown in Table 1 and Fig. 3A. The 20–20 PFG (transmitter) emits a low-intensity AC EM field and establishes a cube or dome-shaped VoM ranging up to a maximum of 66 cm (Aurora v3.1 user guide, NDI, Waterloo, ON, Canada); see Fig. S1A for details. The one-sided dome-shaped VoM, which is relocatable through a mechanical arm, makes the system less susceptible to nearby EM interference and suitable for various clinical applications, such as surgical navigation. The system returns the position and orientation of each receiver at 40 Hz in a coordinate frame centered on the transmitter or another receiver. In addition, the system provides a metric called *error-index* for each sample as a measure of sample quality. The error-indices depend on several factors influencing the receiver position estimation, such as EM interference caused by external sources, incorrect system setup, approaching the VoM limit, and an error in the stylus due to physical damage. This index ranges from 0 to 1, with 0 indicating the ideal estimation of a position and 1 the worst-quality estimate. The system alerts users in case of an erroneous measurement, enabling them to discard that from the digitization.

The 20–20 PFG was fixed at the backrest of the digitization chair to project the dome-shaped VoM toward the head (model), see Fig. S1B for details. Unlike a single SEU in the Fastrak system, Aurora used separate units for system control and the sensor (receiver) interface (SIU). Two receivers with 6DOF were used—a disk-shaped reference receiver and a probing receiver (stylus). Unlike the Fastrak system, Aurora’s stylus was approx. 20-cm long general-purpose probe with a blunt tip of 3-mm diameter and without an integrated switch. The system was connected via USB to a computer hosting *NDIToolBox*™ software (Aurora V3.1 user guide; NDI, Waterloo, ON, Canada), and position data for the receivers were recorded. Since neither a digitization software is available nor a switch-enabled stylus for the current Aurora system, we could only record the continuous position data. However, digitization was carried out by programmatically detecting clusters in continuous data. The stylus was pointed at each desired location for 3 s, yielding a cluster of approximately 120 tracking points. A custom Python program using *scikit-learn*^38^ identified these clusters using a threshold of 100 points within 1 mm and then averaged the points in each cluster to yield a single location per cluster. These locations were then saved as the digitization points acquired by the Aurora system. Figure 3B depicts the continuous tracking data points (grey) for thirty-two EEG electrodes and three fiducial locations, whereas Fig. 3C shows the detected digitized point locations (red points). These points were collected from the EEG cap worn over a Styrofoam head model but overlaid on a human scalp model for a better three-dimensional illustration.

### Test models

**Figure 4:**
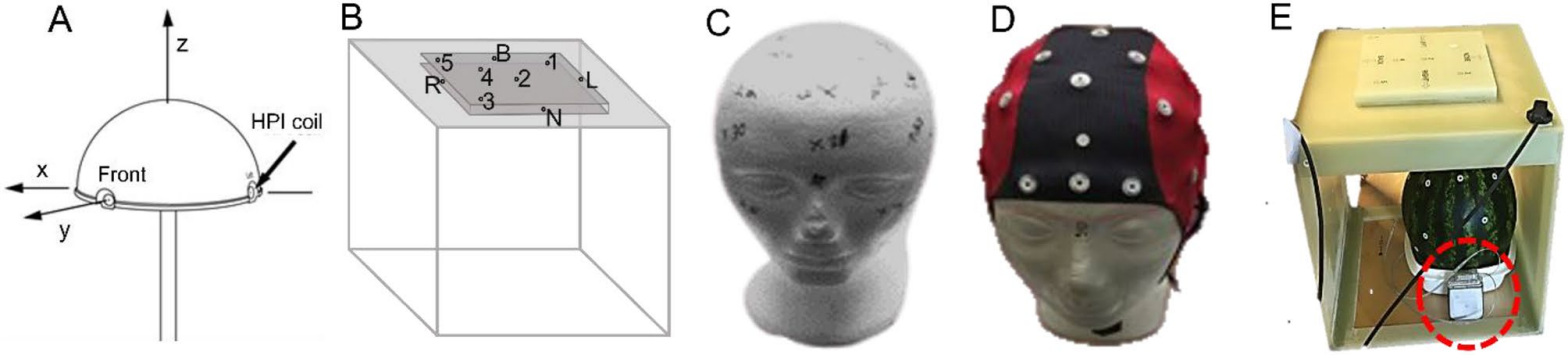
Models used for testing digitization accuracy. (**A**) Spherical MEG phantom, (**B**) 3D test frame, (**C**) Styrofoam head model with 35 marked points, (**D**) EEG cap with 32 electrodes, (**E**) Setup for the digitization test with an active DBS device (red dotted circle).

For examining the digitization performance, we used a spherical MEG phantom, a 3D digitization test-frame, and two Styrofoam head models, one with a set of predefined points and another with an EEG cap. The former two models have predefined points with submillimeter precision, whereas the latter two were carefully digitized with Fastrak TX2 for the predefined points and EEG electrodes to get reference locations for further analysis. We also used a customized model to test the effect of an implanted medical stimulator on digitization. Figure 4A–E shows the test models used in the study. We did not include a human subject for testing the digitization accuracy to avoid the influence of skin and hair softness^32^.

### MEG phantom

A spherical MEG phantom (MEGIN Oy, Espoo, Finland) is commonly used for routine tests of digitizer accuracy at MEG sites. To test the digitization, the phantom has three fiducial points and 4–5 head position indicator (HPI) coil locations; three HPI coils are located at the three fiducial points (Fig. 4A). As a result, the phantom offers five distinct locations for digitization testing. The phantom also has 32 current dipoles at precisely known locations, allowing users to test MEG system performance in source localization.

### 3D digitization test frame

The three-dimensional digitization test-frame (Innokas Medical Oy, Helsinki, Finland), hereafter referred to as the *3D test frame*, is used only to test the accuracy of a digitizer and thus it does not have any source of MEG signals. It is a rigid and hollow cubical fiberglass structure. On top of the cubical frame, a 150-cm elevated fiberglass plate with 16-cm side lengths has nine points excavated 2-mm deep at precisely defined locations (Fig. 4B). The first four points, labeled Left (L), Nasion (N), Right (R), and Back (B), are in the center of the four sides of elevated plates; the following five points are on the upper face. None of the points overlap, so the frame offers nine distinct points for digitization tests.

### Styrofoam head model with marked points

We used 35 marked points on the “scalp” area (32 at random positions and three fiducials (nasion, left and right pre-auricular points), on a Styrofoam head model with a 52-cm circumference. This model mimics scalp digitization without an EEG cap, which is often the case in MEG acquisition, or digitization of individually-placed EEG electrodes (Fig. 4C). The points were meticulously digitized with the standard Fastrak TX2 system, by placing the model 15 cm far from the transmitter. The positions were saved in the MEGIN head coordinate system as a reference for further testing with the model.

### Styrofoam head model with EEG cap

To assess the systems' performance during EEG electrode digitization, we digitized a 32-channel EEG cap (ANT Neuro, the Netherlands) worn on another Styrofoam head model with a circumference of 61 cm (Fig. 4D). We meticulously digitized the electrodes and three marked fiducial points using the Fastrak TX2 system by placing the model 15 cm away from the transmitter. The positions were saved in the MEGIN head coordinate system as a reference for further testing with this model.

### 3D test frame with deep-brain stimulator

DBS (deep brain stimulator) is a typical stimulator in neurological patients. DBS electrodes are implanted in the brain, and the control unit and the wires may interfere with the transmitter field. To evaluate the effect of a surgical implant on digitization, we inserted the two electrodes of a DBS (Activa PC; Medtronic, Dublin, Ireland) into a watermelon placed inside the 3D test frame. The electrodes were ~ 8 cm deep and separated by ~ 4 cm. The arrangement is shown in Fig. 4E, the nine points of the 3D test-frame were used to test digitization while operating the DBS in different modes.

### Ethics statement and informed consent

No human data were recorded in this study, and all the tests were performed on non-living test models. The scalp model depicted in Fig. 3B,C was obtained from a publicly available dataset^39^, which was recorded during our previous study^40^. Written informed consent for using the scalp model in the current study and publication was obtained from the participant.

### Measurements and data collection

Data from all three EMT systems were collected to examine tracking fluctuation, digitization accuracy, and reproducibility. The tracking fluctuation shows the intrinsic property and robustness of the EMT systems in position tracking. However, in MEG/EEG studies, since the system is used for digitization rather than continuous position tracking, submillimeter digitization accuracy is crucial for accurate transformations and estimating a precisely warped template MRI. Therefore, an extensive evaluation of digitization accuracy in the presence of several known and potential influencing factors was done. *PiMgr*™ (Polhemus Inc., Colchester, VT, USA) and MEGIN’s digitization software *Isotrak server* were used to record data from the two Fastrak systems. Data from the Aurora system were recorded using *NDIToolBox*™ software (NDI, Waterloo, ON, Canada). All recordings were taken one by one from the three systems, and no other transmitter was active during measurements. Each transmitter was fixed to the backside of the wooden digitization chair, ~ 110 cm above the floor and more than 150 cm below the ceiling. We ensured that the transmitter and receiver cables did not contact one another or the floor until intentionally tested for such conditions. Furthermore, no magnetic or electrically conductive object was within a 150-cm range of the transmitter unless it was explicitly introduced for a test condition.

### Position tracking fluctuation

An EMT system continuously tracks each active receiver and accumulates and outputs the location data in different ways based on application requirements. In MEG/EEG digitization, for example, a set of continuous data samples from the stylus can be digitized using event-marking and averaging or spatial clustering. If a receiver remains fixed, an EMT system should ideally return the same coordinate over time. However, measurements from all EMT systems exhibit some variation, referred to as *position tracking fluctuation*^33,34^, which depends on transmitter field, system resolution, transmitter–receiver distance, and ambient interference. A larger tracking fluctuation may introduce spatial inaccuracy when converted to digitization points. Therefore, a robust EMT system should have minimum tracking fluctuation. We measured the tracking fluctuations by varying the transmitter–receiver distance and changing the ambient interference. To investigate the native (with no ambient noise) tracking fluctuation, the stylus tracking data were recorded in the global coordinate frame by fixing the stylus tip at 5, 15, 25, 35, and 45 cm away from the transmitter. Furthermore, to record the change in the position tracking fluctuation due to EM interference, the data were collected by fixing the stylus at 15 cm and swinging a magnetic object at approximately 5, 15, 25, 35, and 45 cm away from the transmitter. These objects were: 18-×-25-cm^2^ copper plate, 2-×-2-cm^2^ copper plate, loop made of copper loop, key set, golden jewelry, spectacles with a metallic frame, electronic calculator, credit card, coiled (spiral) cable, board pin, permanent magnet, mobile phone, metallic paper clip, Bluetooth mouse, and steel scissors. The details of these objects are listed in Table S1 and they are shown in Fig. S2.

### Effect of transmitter–receiver distance and movement

The distance of head digitization points may vary between 5 and 40 cm from the transmitter, depending on the patient’s height. Since the transmitter field decays over the distance from the transmitter, the accuracy deteriorates with distance^32^. To find the optimal VoM, we, therefore, measured the field strength of the three transmitters with a spectrometer and observed that the field strength drops approximately by a factor of 10 at ~ 40 cm from the transmitter. Thus, the possibility of higher digitization errors would increase when we moved farther away from the transmitter, especially beyond 40 cm. To investigate the effects of transmitter–receiver distance, we tested the digitization accuracy of all three systems by digitizing the 3D test-frame while it was kept still at 15, 25, 35, and 45 cm from the transmitter. In addition, the same measurements were taken at these four distances by moving (rotating and tilting) the frame during digitization to estimate the effect of (head) movement during MEG/EEG digitization.

### Effect of cable contacts

Engels and colleagues^32^ reported a potentially minor increase in digitization error due to physical contact between transmitter and receiver cables of the Fastrak TX2 system; however, they found high digitization errors in a few test cases. Also, MEG users had anecdotally reported higher digitization errors when the cables were warped around or laid over a steel-reinforced concrete floor. Since the Fastrak and Aurora cables are physically different, the effects of physical contact between cables are also expected to differ. Therefore, we extended our investigation to determine how effectively the cable designs (shielding and inner architecture) cancel out such interference. We created conditions that users commonly face while handling the cables during digitization, such as transmitter–receiver cable contacts, cables contacting the floor, and the cables wrapped around (twisted together). The data from the 3D test frame were collected in these conditions with all three systems by placing the frame immobile at 15 cm from the transmitter.

### Effect of nearby electromagnetic interference

A magnetic object in the vicinity may interfere with the transmitter field, lowering the digitization accuracy. To evaluate such effects, the transmitter field was disrupted by external EM fields of different strengths during digitization. The 3D test frame was digitized at 15 cm from the transmitter, while various objects were present: a tiny object such as a small copper plate or loop; a significantly large object such as an 18 × 25 cm^2^ copper plate or a mobile phone; or a prevalent infrastructural source of potential artifacts such as a metal cabinet or a steel-reinforced concrete wall. The first four objects were separated by 15, 25, 35, and 50 cm, while the latter two were separated by 25, 50, 75, and 125 cm. Further, to investigate the effect of medical implants, the model replicating a DBS implant, as shown in Fig. 4E, was digitized at 15 and 35 cm far from the transmitter while operating the DBS in various therapy settings.

### Effect of transmitter’s field on MEG

Because the MEG sensor array (probe unit) is usually located in a magnetically shielded room (MSR) and the digitization is performed outside of the MSR, the EMT transmitter field is not considered to interfere with MEG recordings. However, in a compact lab layout, the transmitter can be close to the MSR wall and its residual field inside could be non-negligible. Therefore, it is critical to determine whether an active transmitter can generate an artifact in an ongoing MEG recording. To investigate such an effect, we recorded MEG with a 306-channel TRIUX™ system housed in a lightweight MSR (TRIUX™; MEGIN Oy, Espoo, Finland) while having an active transmitter around the MSR in five different positions. For each of the three systems, a transmitter was—(i) kept still ~ 30 cm outside the MSR wall with the MSR door closed, (ii) kept swinging outside near the projector aperture with the MSR door closed, (iii) kept still inside the MSR ~ 200 cm from the MEG sensors with MSR door closed, (iv) kept outside close to the MSR door with the MSR door closed, and (v) kept outside close to the MSR door with the MSR door slightly open (Fig. S3A). 2-min continuous MEG data (in the absence of a subject) were recorded for each of the three systems, for those five positions of the transmitter. In addition, 2-min reference data were also recorded for each system, without an active transmitter around the MSR. The datasets were recorded at a 1-kHz sampling rate and without using Internal Active Shielding^41^.

### System-specific effects on source localization

Finally, to evaluate the effect of digitization with each of the three systems on source localization, we recorded MEG from a phantom by sequentially activating eight current dipoles with 1000-nAm peak amplitude. First, the same phantom was digitized by each EMT system by positioning it 15, 25, and 35 cm from the transmitter. Then, we replaced the digitization information from the phantom MEG file with the digitization data at three distances for each of the three systems. Thus, we obtained three sets of phantom MEG data for each system, including the fiducial and HPI locations digitized at 15, 25, and 35 cm.

### Data analysis

The position tracking fluctuation, digitization accuracy, and effect of several noise sources on digitization for all three systems were evaluated. The mean and quartile values (Q1, Q2, Q3) were computed and the results were visualized as overlapped box–violin plots. A violin plot’s width on the y-axis is proportional to the data distribution at that point. Mean, IQR (inter-quartile range Q3–Q1), outlier percentage, and extreme percentage were computed for each plot. The thresholds for outliers and extremes were determined using 1.5 × Q3 and 3.0 × Q3, respectively. We also performed statistical tests to find differences between the results from the three systems.

### Position tracking fluctuation

For each of the three systems, the position tracking fluctuation was computed in the global coordinate system (wrt. Transmitter origin) for each transmitter–receiver distance. The mean position tracking fluctuation, ${mPTF}_{d}$ was computed as the difference between consecutive points:

1 $${mPTF}_{d}=\frac{1}{n}{\sum }_{k=1}^{n-1}{[{\left({x}_{k+1}-{x}_{k}\right)}^{2}+{\left({y}_{k+1}-{y}_{k}\right)}^{2}+{\left({z}_{k+1}-{z}_{k}\right)}^{2}]}^\frac{1}{2}$$

where (${x}_{k}$*, *${y}_{k}$*, *${z}_{k}$) is the cartesian coordinate of the *k*th point, $n$ is the total number of tracking points, and the subscript $d$ stands for the transmitter–receiver distance.

### Digitization accuracy

Both the Fastrak and Aurora systems provide position and orientation information for each connected receiver, but only position data are utilized in MEG and EEG digitization. The error in digitizing any point was computed as the Euclidian distance between the actual location (${x}_{act}$*, *${y}_{act}$*, *${z}_{act}$) of the point and the location estimated by the digitizer (${x}_{est}$*, *${y}_{est}$*, *${z}_{est}$), in the same coordinate frame. An accurate digitizer should yield minimum digitization error, and for MEG or EEG it should be in the submillimeter range. The mean digitization error ${mE}_{d}$ for transmitter–receiver distance was computed as

2 $${mE}_{d}=\frac{1}{n}{\sum }_{k=1}^{n-1}{[{\left({x}_{act}-{x}_{est}\right)}^{2}+{\left({y}_{act}-{y}_{est}\right)}^{2}+{\left({z}_{act}-{z}_{est}\right)}^{2}]}^\frac{1}{2}$$

where the subscript $d$ represents the transmitter–receiver distance, (${x}_{act}$*, *${y}_{act}$*, *${z}_{act}$) is the cartesian coordinate of the *k*th point, and $n$ is the total number of tracking points. To evaluate the system’s digitization accuracy, we measured the digitization error for the points with precisely known locations on the accurately defined rigid frames—the spherical MEG phantom and the 3D test frame. Since the blunt tip of the Aurora stylus could not reach the very end of the narrow points on the 3D test frame, its measurements were compensated by subtracting the point depth (2 mm) from their normal directions.

### Source localization

We fitted ideal current dipoles to the phantom data to investigate how the transmitter–receiver distance affects the source reconstruction through imperfect co-registration. Using the Source Modelling Software (MEGIN Oy, Espoo, Finland), we localized all eight current dipoles from the three sets of phantom MEG data with digitization performed by the Fastrak TX2 system at transmitter–receiver distances of 15, 25, and 35 cm. We repeated the same dipole localization on the MEG data with digitization points acquired by Fastrak TX1 and Aurora systems. Further, for each case, we calculated the dipole localization errors with respect to the true locations and compared the distribution against a *control* set of localization errors, which was determined by taking the mean of localization errors over the ten previous routine phantom tests, digitized using Fastrak TX2 at ~ 15 cm from the transmitter. We also compared the localization errors from data recorded using Aurora and Fastrak TX1 against the ones with Fastrak TX2 at the corresponding distance from the transmitter. We used Welch's two-tailed t-tests to perform the statistical comparison.

## Results

We collected the results from the position tracking fluctuation, digitization accuracy, and source analysis for the three digitization systems as box–violin plots. The table attached under each plot shows the statistics, where the first row labels the data category, and the second onward shows the mean value, IQR, outlier percentage, and extremes percentage, respectively. The smaller the IQR value, the lower the fluctuation is and, therefore, better performance. The black and red markers (circle) in the violin plots represent the outlier and extreme values respectively. For all the violin and scatter plots, mint, lavender, and coral colors represent results from the Fastrak TX2, Aurora, and Fastrak TX1 systems, respectively.

### Position tracking fluctuation

**Figure 5:**
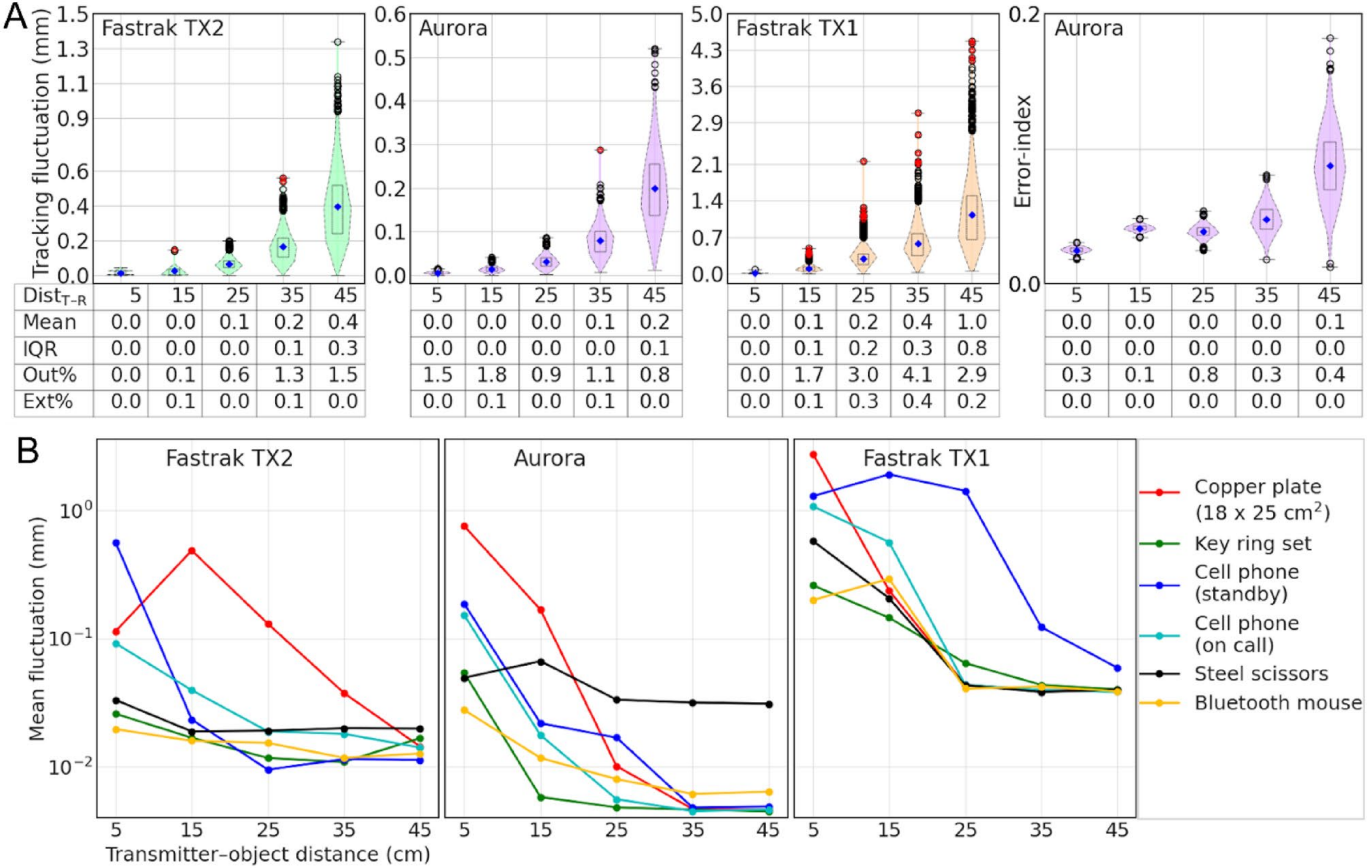
Position tracking fluctuation of the three systems (**A**) native fluctuations are shown in the first three subplots, and the fourth subplot shows the error-index provided by the Aurora system, (**B**) mean fluctuation when an interference source was at various distances from the transmitter.

Figure 5A depicts the native tracking fluctuations of the EMT systems, which increase with transmitter–receiver distance. The Fastrak TX2 system shows tracking fluctuation below 0.5 mm if the receivers remain within a 40-cm range in the VoM. However, Aurora offers an extended range with position tracking fluctuation below 0.5 mm. On the other hand, the Fastrak TX1 shows a higher tracking fluctuation beyond 20 cm from the field origin. The values from Fastrak TX1 were presented on a separate Y-scale to visualize the distribution better and to ease the interpretation. In the Aurora error-index plot, the increasing values with distance demonstrate its use for effectively filtering out low-fidelity tracking data. We found that when the stylus was kept at 15 cm, and objects of everyday use were kept at specific distance ranges from the transmitter, only six out of the seventeen objects showed a mean fluctuation higher than 0.2 mm; see Fig. S4 for details. Figure 5B demonstrates the results for those showing a mean fluctuation higher than 0.2 mm at least at one of the five distances. The plots show increases in tracking fluctuation when the noise source is kept closer to the transmitter. The mean fluctuation for the tested objects would stay within 0.1 mm for all three systems if the objects were kept beyond 35 cm from the transmitter. Placing a magnetic object closer to the transmitter perturbs the field more which increases the fluctuation. Fastrak TX2 seems quite noise-resilient and shows the tracking fluctuation below 0.1 mm until an object with a strong magnetic field, such as a large metal plate or a mobile phone, is brought within the 25-cm range of the transmitter TX2. Until a potent source of interference, such as a cell phone or large metal plate, was kept within 20 cm of the transmitter, the Aurora system maintained its low tracking fluctuation (< 1 mm). The interference appears to have the biggest impact on the Fastrak TX1 system.

### Effects of transmitter–receiver distance and movement

**Figure 6:**
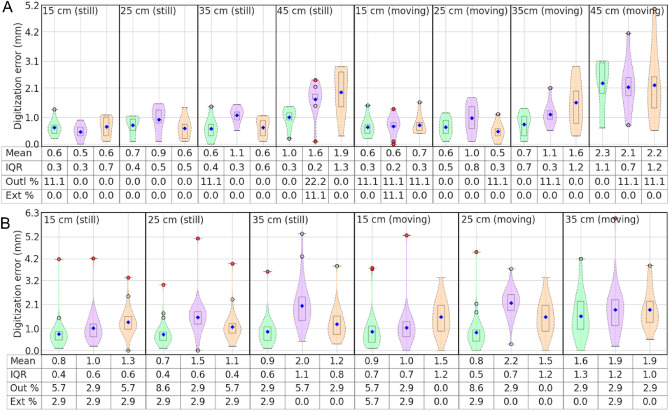
Digitization error for the (**A**) 3D test-frame and (**B**) Styrofoam head model with 35 marked “scalp” points, when models were kept either still or moving during digitization and when the transmitter–stylus distance was varied from 15 to 45 cm.

The effects of transmitter–receiver distance and subject’s head movement on digitization were examined in terms of digitization error; a higher error indicates a lower digitization accuracy. The digitization error for a point was estimated as the Euclidian distance between its actual (or reference) and estimated positions in the MEGIN head coordinate frame. Figure 6A,B shows the digitization error distribution for the rigid 3D test frame and the marked Styrofoam head model. Note that one point (2.9 percent of the total) in the occipital region of the head model was in such a position that the chair's backrest caused difficulty during digitization, resulting in an extreme error for all tests (Fig. 6B). Overall, the results show a positive relationship between digitization error and transmitter–receiver distance as well as test model (head) movement. The digitization error distribution for the 32-channel EEG cap also showed a similar pattern (Fig. S5).

The mean and IQR values for all three systems showed reliable digitization when the test models were kept still, with the current digitizer Fastrak TX2 performing slightly better, and the mean digitization error remained below 1 mm within 40 cm. This 'safe' range decreased to less than 35 cm when the test model was moving during digitization. Please note that the 3D test-frame with nine rigid and precisely defined point locations represents an ideal test case for digitization. In contrast, the Styrofoam head model has some skin-like softness, so it describes a more practical situation for head digitization with slightly higher digitization errors^30^. The other two systems, Aurora and Fastrak TX1 showed marginally higher digitization error and IQR than the Fastrak TX2 when transmitter–receiver distance raises higher than 35 cm. This effect was due to the receivers approaching the edge of the VoM. Utilizing the tracking samples with higher error-indices, the digitization error in the Aurora system could be reduced to improve its accuracy. Also, unlike the Fastrak stylus, the 2-mm-thick stylus from Aurora could not provide a good probing resolution for the smooth Styrofoam model. Furthermore, the mean and IQR values for the five identically digitized cases in Fig. 6A (still at 15, 25, and 35 cm, and moving at 15 and 25 cm) varied by less than 0.5 mm, indicating a high level of digitization reproducibility (or repeatability) if digitized within 35 cm from the field origin.

### Effects of cable contacts

**Figure 7:**
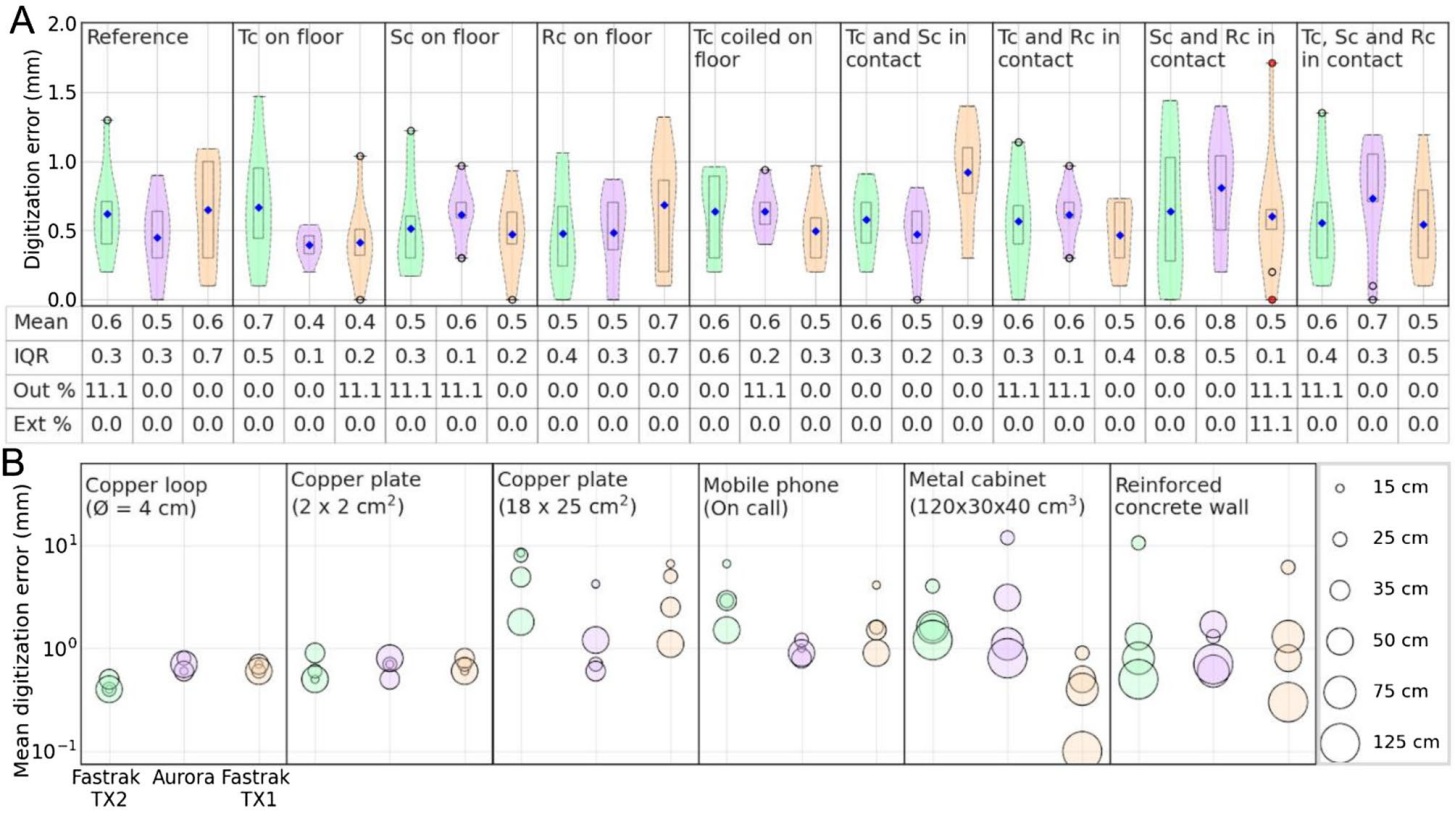
Digitization error when using the 3D test frame with (**A**) transmitter (Tc), stylus (Sc), or reference receiver (Rc) cables in various positions; and with (**B**) interfering objects at varying distances from the transmitter; the marker size encodes the transmitter-to-object distance.

Figure 7A depicts the error distribution for digitization of the 3D test frame at 15 cm from the transmitter when the transmitter cable was in contact with a receiver cable or a reinforced concrete floor. The mean digitization error for all the systems in all the conditions stayed below 1 mm; however, the maximum error and IQR are sometimes higher than the reference/control condition (in Fig. 6A when frame kept still at 15 cm). Although there is no notable systematic error, the higher IQRs show increased variability or reduced stability in position estimation, potentially due to increased fluctuation in position tracking.

### Effect of nearby electromagnetic interference

Figure 7B summarizes the effect of interference on digitization accuracy when the 3D test frame was digitized at 15 cm from the transmitter in the presence of a magnetic object at a known distance; see Fig. S6 for detailed violin plots. We observed that the mean digitization error in the presence of small magnetic objects stayed below 1 mm, indicating a minimal disruption in the EM field and thus position estimation. Fastrak TX2 showed comparatively lower digitization error, except for an outlier or extreme in a few cases. However, objects with large magnetic interference, such as a large metal plate or a mobile phone, resulted in a notably high digitization error even at the distance of 50 cm. In such situations, Fastrak TX2 displayed a high digitization error. This could be the result of such objects being exposed to TX2's strong AC electromagnetic field, which created a high eddy current that caused a strong field distortion^35^. On the other hand, unless a significant interference source was extremely near to the transmitter, Aurora and Fastrak TX1 were impacted less likely due to their smaller VoM. When clustering continuous tracking data, the Aurora system's digitization program eliminated "poor" tracking samples since it used clustering with a spatial limit of 2 mm. However, the Fastrak TX2 and TX1 did not reject any data for digitization based on spatial thresholding. As a result, despite TX1 having a smaller VoM, Aurora displays a lower mean and IQR for the digitization error. When testing the digitization with nearby infrastructural sources of potential artifacts, such as steel-reinforced concrete walls and a large metal cabinet, we found a large increase in the digitization error if the disturbance sources were close (< 50 cm) to the transmitter. Fastrak TX2, due to its strong and extended VoM, showed a mean digitization error > 1 mm, even if such a noise source is located within 125 cm.

The effect of an implanted DBS on digitization is plotted in Fig. S7. Due to the unavailability of the TX1 transmitter during the DBS tests, we could only take measurements with Fastrak TX2 and Aurora systems. The digitization error distribution for both systems showed that the mean error stayed within 1 mm for all operating modes when the test-frame was 15 cm far from the transmitter. However, when increasing the distance from 15 to 35 cm, the mean error for Fastrak TX2 slightly increased but stayed within 1.5 mm with a few outliers. The error for the Aurora system was found to be slightly higher at 35 cm. Overall, the increasing digitization error seems to be due to the distance rather than the presence of the DBS.

### Effect of transmitter’s field on MEG

The power spectral density (PSD) of MEG data was computed for the three transmitters and compared to the PSD of the reference data. The data were preprocessed with the Signal Space Separation method (SSS)^42^ using MaxFilter™ software (MEGIN Oy, Espoo, Finland). We found no abnormalities in the MEG data when comparing the PSDs until the transmitter was deliberately kept within the MSR or just outside the MSR door with the door open. Detailed results are shown in Fig. S3B.

### System-specific effects on source localization

**Figure 8:**
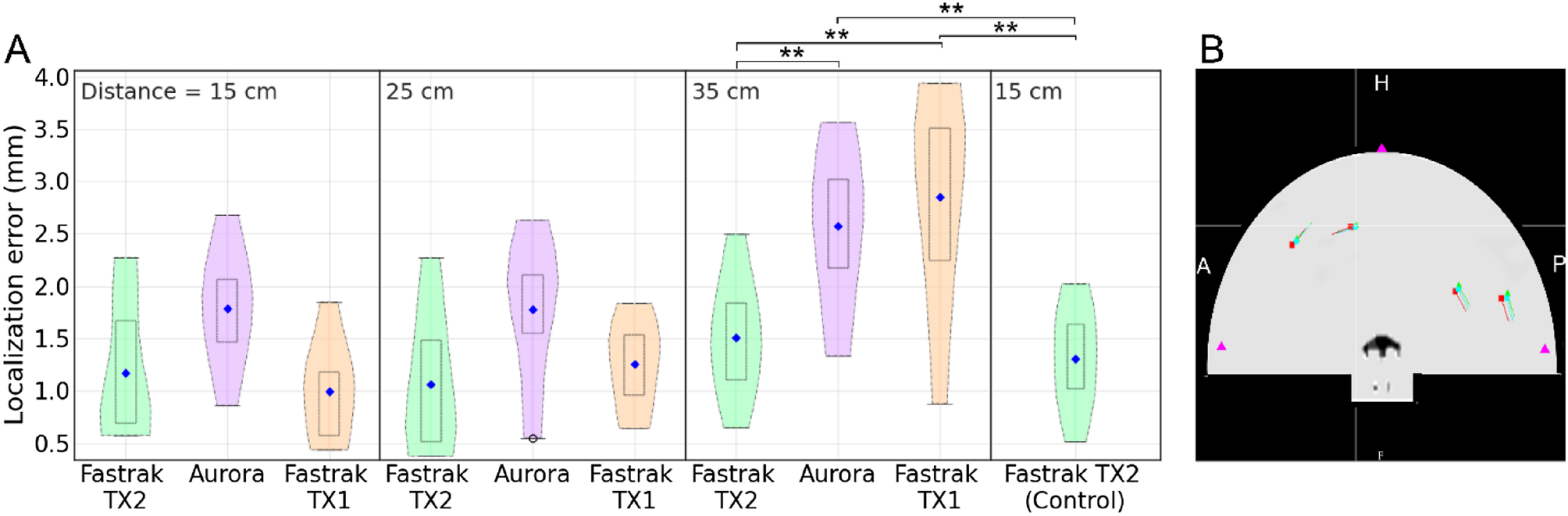
(**A**) Localization error for the eight dipoles, (**B**) the estimated position and orientation of four of the eight dipoles, when the digitization was done using the three different systems. The three magenta triangles represent the location of the HPI coils in the anterior (A), superficial (H: head), and posterior (P) directions.

Figure 8A shows the distribution of source localization errors for the eight dipoles where the digitization was done using one of the three EMT systems at specific distances from their transmitter. The error distributions were compared against the *control* that was computed by taking the mean over ten routine phantom tests. In comparison with the control set of error distribution, we observed significant differences (*p* < 0.05) for Aurora and Fastrak TX1 at 35 cm. When these two systems were compared against Fastrak TX2 errors at either of the three distances, they also showed significant differences (*p* < 0.05). We also observed an insignificant difference for Aurora at 25 cm against the control (*p* = 0.056). Figure 8B visualizes the difference in the estimated position and orientation for four of the eight dipoles overlaid on the CT image of the phantom. The p-values show that until the digitization was done beyond 25 cm, the dipole fitting was not significantly different from the control measures and the localization errors stay within 3 mm which is quite acceptable. The distribution shows that the source localization error increases with increased transmitter–receiver distance during digitization.

## Discussion

The study investigated several performance traits of the three EMT systems for digitization under diverse real-world circumstances. The tracking fluctuation was found to be increasing with increasing transmitter–receiver distances. The native tracking fluctuation of the Aurora system was found lower than that of the Fastrak® systems, indicating higher stability in position tracking. All three systems showed a mean fluctuation of less than 0.5 mm unless either a strong disruption was introduced to the transmitter field or the transmitter–receiver distance was increased beyond 40 cm. The Fastrak TX2 system was found highly tolerant to the effects caused by the magnetic objects placed in the VoM due to the strong field generated by the TX2 transmitter. The Aurora and Fastrak TX1 systems also showed good tracking stability and robustness against the noise sources, unless the measurement was taken more than 40 cm from the transmitter or a strong EM distortion was present closer than 25 cm. The Fastrak TX1 system sometimes showed even higher tolerance against nearby noise sources, possibly because of its shorter field (VoM).

We found that none of the three systems added any artifacts to an ongoing MEG recording until an operating transmitter was purposefully kept inside the MSR or used extremely close to the open MSR door. However, we recommend having at least a one-meter distance from the MSR to the transmitter and receiver for avoiding distortion to the digitization. We did not see a systematic increase in digitization error due to the transmitter or receiver cables laying on the floor, nor due to physical contact or twisting of the cables; however, the maximum error and IQR were sometimes higher than the control condition. Therefore, it is recommended to avoid such contacts or having the cables on the steel-reinforced floor or large metallic structures.

Generally, we observed that the digitization accuracy drops with increasing distance between the transmitter and receiver. Therefore, it is crucial to perform the digitization as close to the transmitter as possible. In the case of a still frame/head model, the digitization error remains lower than that of the moving conditions at the corresponding distances. Since the transmitter field drops by more than a factor of ten at a distance of ~ 40 cm, the digitization accuracy also drops and may result in unreliable digitization. Since a reference receiver is attached to the head during digitization, its position estimation and movement compensation are also compromised when going far from the transmitter. Therefore, the increasing transmitter–receiver distance reduces the accuracy in two ways when digitizing a moving head. Due to smaller VoM, the accuracy of the Aurora and Fastrak TX1 systems drops suddenly after ~ 40 cm. The IQR in the case of still frame digitization was found lower than in the corresponding moving cases, and it showed a sudden increase after ~ 40 cm. Therefore, while digitizing a non-cooperating subject, for example, in the case of pediatric MEG/EEG, the experimenter should ensure that the subject’s head remains closer than about 35 cm to the transmitter.

All three systems can tolerate moderate interference sources to the transmitters’ field. In the case of strong sources such as a large copper plate inside the VoM, the stronger and more extended field of the Fastrak TX2 system can interfere with and compromise the digitization accuracy. The other two systems benefit from the more compact field, but the digitization error jumps high if the interference source is too close to the transmitter. Therefore, Aurora and Fastrak TX1 systems sometimes showed comparatively lower digitization errors during the noise tolerance tests. In such cases, the Aurora also gets the benefit of its error-indexing feature, but when a strong interference is closely placed, the number of points with a higher error-index increases. Some of these erroneous points get included in digitization due to the current approach of clustering-based digitization and may deviate from the true location. Overall, Fastrak TX2 performed better with less than 2 mm of digitization error unless a strong noise source was less than 50 cm from the transmitter. All three systems were found quite robust against large construction-based ambient noise sources such as steel-reinforced walls and metal cabinets, as long as they were at least 100 cm away. Thus, users should maintain a safe distance of ~ 120 cm from such interfering structures when using Fastrak TX2. Finally, we did not observe any substantial effect of an operating DBS on digitization accuracy.

Source localization of the phantom dipoles showed that the use of the three digitizers resulted in a small difference in the localization of the dipoles. Digitization with Fastrak TX2 yielded a localization error below 2 mm for all the test distances, indicating that the Fastrak TX2 system is sufficiently accurate and robust for digitization in MEG/EEG applications. The error when digitized with Aurora or Fastrak TX1 systems within a 30-cm range also stayed < 2 mm but increased to 3 mm when digitized at 35 cm. Overall, the localization error stayed below 5 mm, which may still be acceptable in most of the MEG/EEG studies. Thus, Aurora and Fastrak TX1 can also be considered as potential digitizers with a restricted VoM radius of under 40 cm for reliable digitization. The Fastrak TX2 was found to be the most accurate; however, the 150-cm-radius VoM is large, and it can be difficult to get such a large area free of magnetic or conducting objects in a compact MEG site. The TX1 transmitter, on the other hand, provides a much smaller VoM. In the Aurora system, the 20–20 PFG provides a usable planar magnetic field up to approximately 55 cm from the transmitter, which is sufficiently large for the digitization of most human participants. However, an approximately 20% increase in the VoM would help users to cover all the age groups of human subjects by fixing the transmitter in one position (behind the digitization chair). An ideal VoM for MEG/EEG digitization should be large enough to cover the full head of all the human subjects but not too large to interfere with the nearby magnetic objects. A VoM of 70–80 cm from the transmitter origin and uniformly directed toward the patient/subject’s head would be suitable for MEG/EEG digitization. Alternatively, an adjustable transmitter placement would make the system more efficient to cover a larger span of patient heights.

The results from tracking fluctuation, digitization accuracy, reproducibility, and other tests revealed that the Aurora EMT system can also be utilized as a digitizer for MEG and EEG applications, and the real-time error-indices can be utilized to discard erroneous samples and improve the digitization fidelity. However, some modifications would be required to turn the Aurora system into a *ready-to-use* digitizer for MEG/EEG applications; for example, a switch-enabled stylus and a user-friendly digitization software are necessary to avoid the customized clustering method. The tip of Aurora’s stylus should also be thinner than the current one to improve the probing resolution. In addition to utilizing the error-index feature, a real-time voice alarming system would also help the users restrict within the VoM and avoid erroneous digitization.

In the study, the fluctuation test with a Bluetooth mouse showed that all three systems are robust against the possible noise caused by a Bluetooth device if separated by at least 25 cm from the transmitter. Therefore, a wireless stylus can be incorporated with either the Aurora or Fastrak systems to eliminate long stylus cables and improve the systems’ usability. Notably, an additional footswitch replacing the stylus switch should be integrated into the systems to avoid possible shaking while digitizing, especially for the fiducial points.

Since the study includes a large set of evaluations, testing all the cases with a human subject was not possible due to the unavailability of the ground truth (precisely known stable points). Although the majority of the findings are based on rigid objects, the study thoroughly evaluated many practical situations of MEG and EEG digitization, and thus the findings are transferable to human subject digitization. However, factors such as the operator’s hand tremor, stylus orientation, and timing of the stylus button press may also affect the accuracy, and extra care is needed while digitizing a human subject. Skin softness and head movement, particularly in pediatric subjects, may reduce digitization accuracy. For comparing the Aurora system, we replicated all the test cases similar to the Fastrak measurements. However, some differences, such as the long, blunt edge stylus lacking a switch and tracking to digitization conversion due to a lack of digitization software, may have introduced some additional errors. Therefore, the comparison can be improved by using a smaller, switch-enabled stylus with a thinner edge and digitization software that uses the error index. Although optical scanners are becoming more common, especially for high-density EEG systems and OPM-based (optically pumped magnetometer) MEG systems, they face a line-of-sight problem instead of EM interference, and therefore such systems should also be evaluated in the future to find the optimal digitization system.

### Guidelines for MEG/EEG digitization

**Figure 9:**
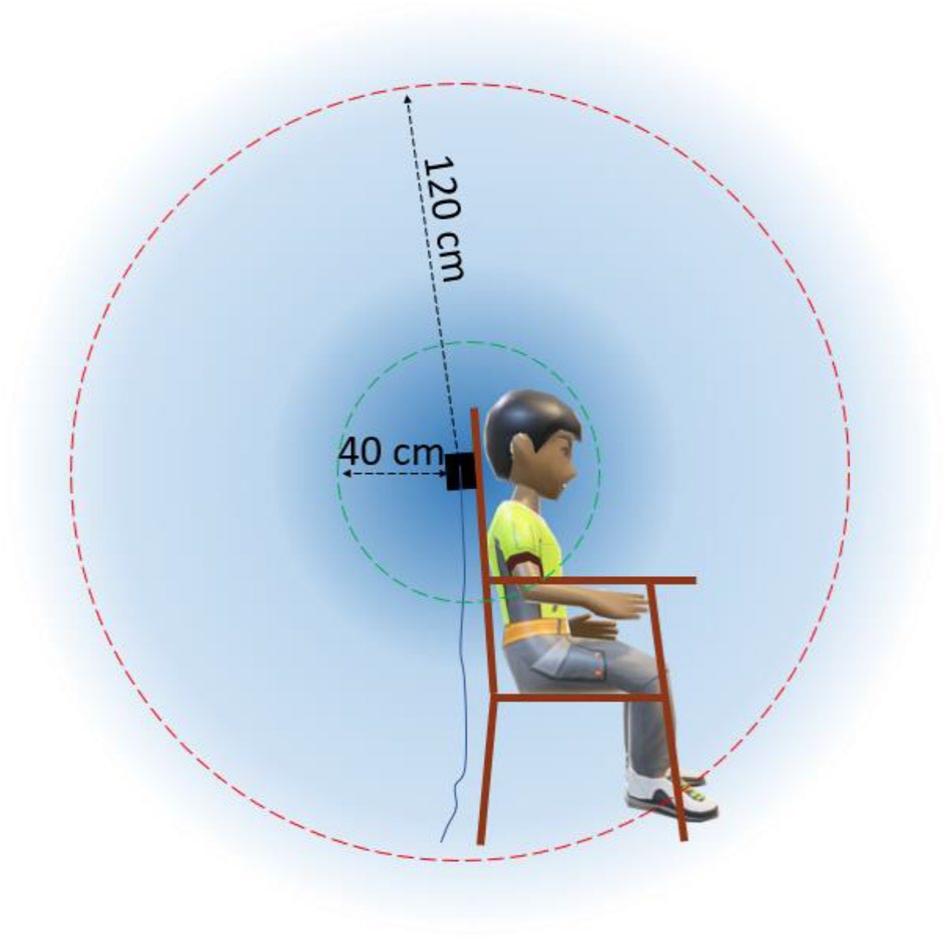
For Fastrak TX2, the green circle (Ø ≈ 40 cm) represents an optimal field (VoM) for digitization, and the red circle (Ø ≈ 120 cm) represents a boundary within which magnetic and conductive objects should be avoided.

According to the results, the following recommendations are made for MEG/EEG digitization using an EMT-based digitizer, especially Fastrak with TX2 transmitter, in order to minimize distortions and to optimize digitization accuracy:

1. The optimal setup for MEG/EEG digitization with a Fastrak TX2 system should be done similar to Fig. 9. Objects with substantial magnetic properties should be avoided within the 120-cm range (red-dotted region). For optimal digitization, the head should stay within the 40-cm range (green-dotted region).
2. The transmitter should be at least 80 cm far from the reinforced floor, roof, and walls to minimize potential interference due to the hidden metal support structures.
3. The distance between the transmitter and the digitization point on the head should be less than 45 cm. For a subject with potential head movement, such as a child, the experimenter should ensure that the subject’s head remains close to the transmitter (< 35 cm), possibly by non-magnetic head rest.
4. The head movement should be slow and minimal (< 2 cm) to improve digitization accuracy.
5. The head-bound reference receiver should not move (shift) more than 1 mm with respect to the head during the digitization process.
6. For the Fastrak systems, the transmitter cable should exit the transmitter module downwards. Physical contact between the receiver and transmitter cables should be avoided.
7. The processing unit (SEU) should be more than 120 cm from the transmitter.
8. The study subject should be thoroughly checked before digitization for any metallic object, phone, hair clip, etc., to avoid distorting the transmitter field.
9. When digitizing a point, the stylus should be kept perpendicular to the surface. To avoid shaking the stylus, an external switch should be used, such as a foot switch, while digitizing the fiducial points.
10. Fiducial points should be defined and reported unambiguously. Although the point where the *Helical crus* touches the *Targus* is usually considered LPA/RPA, some operators use the center of the tragus or the *Intertragal notch* as these two fiducials in their experiments. To avoid adding to co-registration error, the fiducial locations should be explicitly stated, particularly when exporting data to other researchers or to an open-source repository.

## Conclusion

In MEG and EEG, digitization is a crucial step for accurate source imaging. We found that the conventional digitizer (Fastrak TX2) performs accurately and robustly if the following recommendations are considered: (i) Any notable magnetic or conductive material should be avoided within 120 cm of the transmitter; (ii) the head should stay still and be within 45 cm of the transmitter; (iii) physical contact between the system's cables and conductive surfaces should be avoided; (iv) the stylus should be placed perpendicularly to the point when digitizing; and (v) the fiducial points must be clearly defined to avoid possible shifts during MEG-MRI integration. A short-range transmitter (TX1) limits users to a smaller measurement volume and raises the possibility of *mis-digitization*. We found that the popular surgical navigation system (Aurora) could also be used as a digitizer by adding a switch-enabled stylus and a longer stylus cable. An ideal EMT system for MEG/EEG digitization would use a unidirectional planar field with uniform sensitivity until about 70 cm from the transmitter, real-time error estimates, and a footswitch replicating the stylus switch to improve digitizer usability.

## Supplementary Information

Supplementary Information.

## Data Availability

The data collected in the study are available upon a reasonable request to the corresponding author.
