# Supplementary material for: On electromagnetic head digitization in MEG and EEG
Source: Sci Rep. 2023 Mar 7;13:3801. doi: 10.1038/s41598-023-30223-9 (PMC9992397; doi:10.1038/s41598-023-30223-9)
Supplement: Supplementary file 1 — Supplementary Information. [file 41598_2023_30223_MOESM1_ESM.pdf]

# Supplementary information:
# On electromagnetic head-surface digitization in MEG and EEG

Amit Jaiswal[1,2,*], Jukka Nenonen[1], Lauri Parkkonen[1,2]

[1]Megin Oy, Espoo, Finland.

[2]Department of Neuroscience and Biomedical Engineering, School of Science, Aalto University, Espoo, Finland.

*Corresponding author: amit.jaiswal@megin.fi

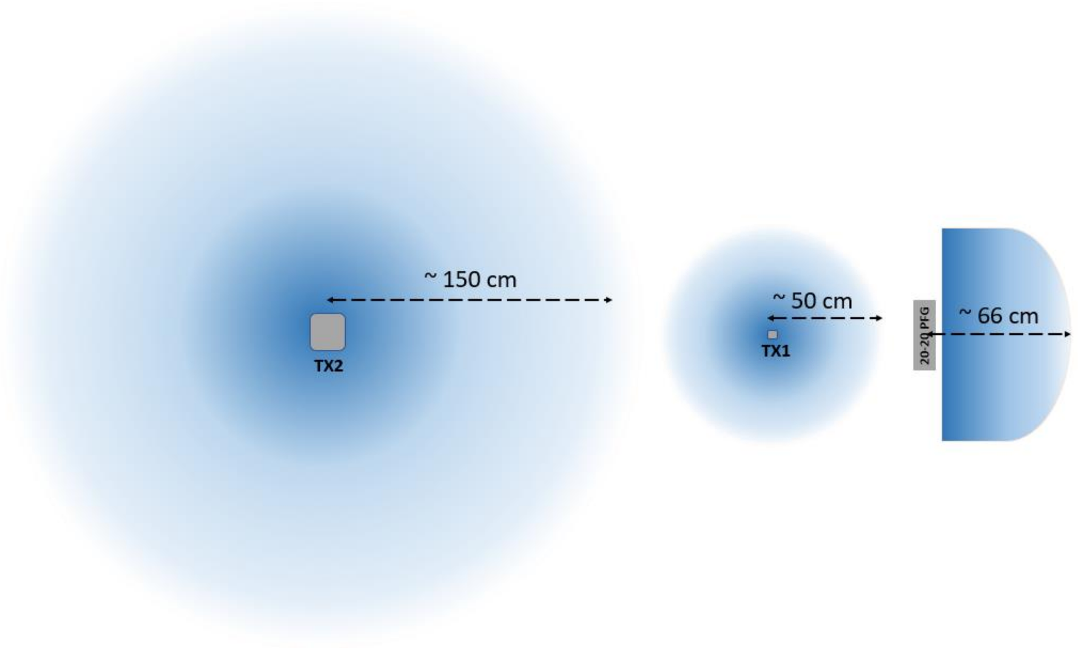


(A)

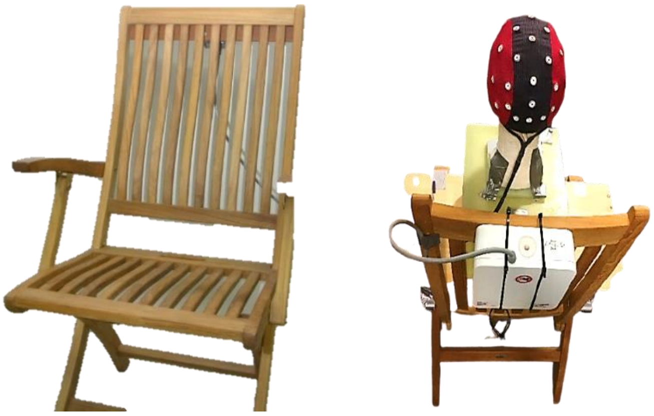

(B)

**Figure S1**. Field pattern. A) Representation of the measurement volume (VoM) for Fastrak (TX2), Fastrak (TX1), and Aurora 20-20 PFG, B) Megin's digitization chair and Aurora 20-20 PFG fixed to chair's backrest during the tests (top view)

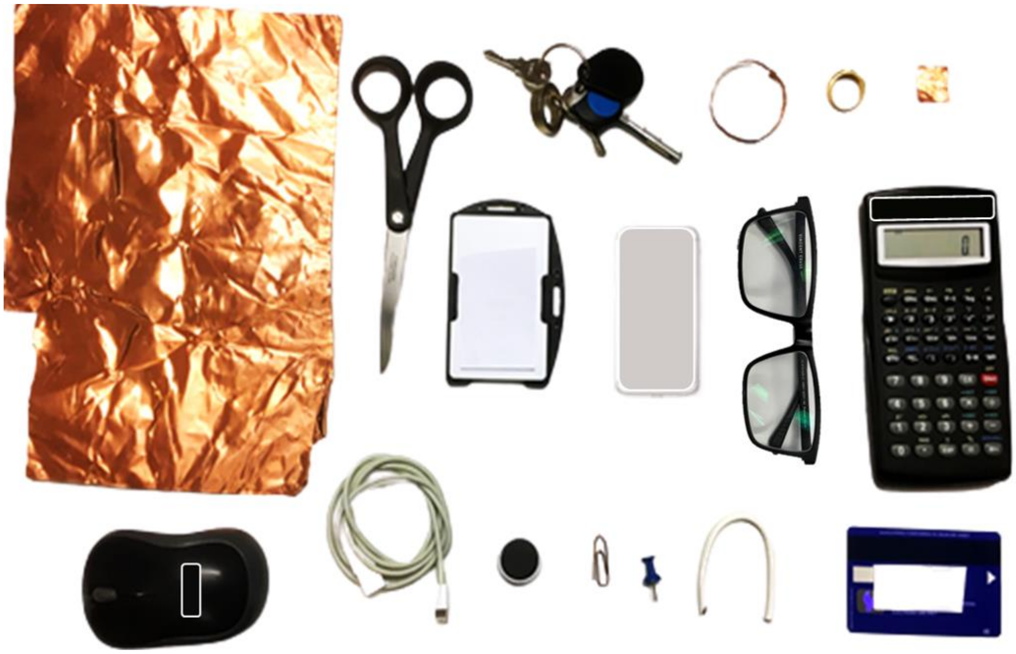

**Figure S2**. The magnetic objects tested during the fluctuation tests of the EMT systems.

**Table S1**. Various magnetic objects were tested during the fluctuation tests of the EMT systems.

| Test object | Size approx. | Specification |
|---|---|---|
| Large copper plate | 18 cm x 25 cm | Thin and flexible |
| Small copper plate | 2 cm x 2 cm | Thin and flexible |
| Copper loop | Ø = 4 cm | Made of 4 mm wide strip |
| Key set | 7 cm | A set of metallic and RFID keys |
| Jewelry | Ø = 2 cm | Golden ring |
| Spectacles with metallic frame | 14 cm wide | Metal frame |
| Electronic calculator | 16 cm x 8 cm x 2 cm | Switched on |
| RFID card | 9 cm x 5 cm | Door card |
| ATM card | 9 cm x 5 cm | With magnetic strip |
| Spiral cable | Ø = 6 cm | phone charging cable |
| Board pin | 1 cm | Iron pin |
| Permanent magnet | Ø = 3 cm | Board magnet |
| Mobile phone | 14 cm x 7 cm x 1 cm | Switch on |
| Paper clip | 3 cm | Made of iron |
| Bluetooth mouse | 10 cm x 5 cm x 3 cm | Switched on |
| Dental brace’s metallic frame | 6 cm | Half loop replicating brace frame |
| Scissors | 15 cm | Stainless steel |

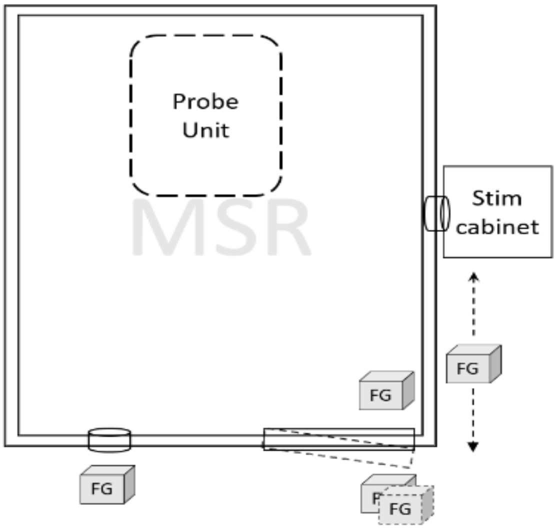


(A)

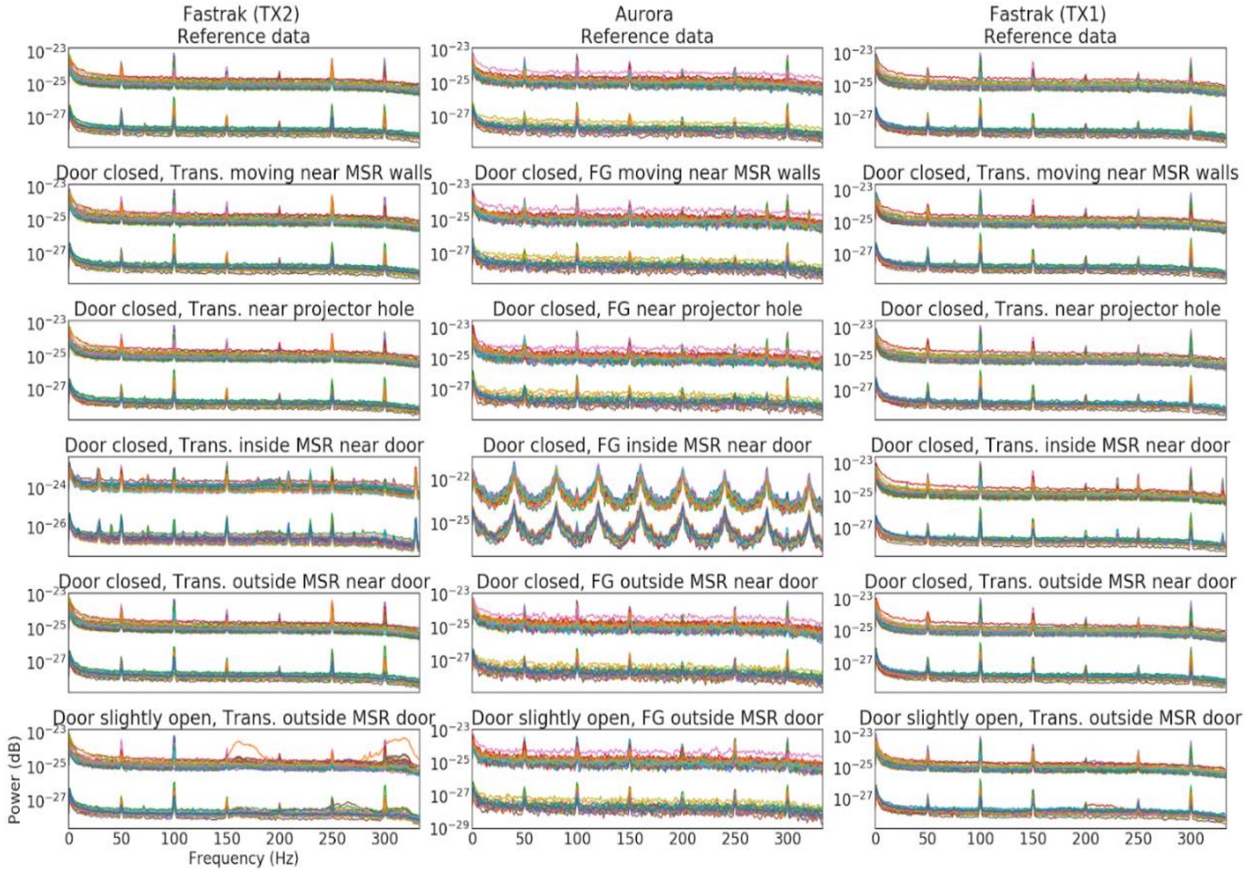


(B)

**Figure S3**. Illustration of the transmitter/FG artifacts in MEG data (A) Positions of the transmitter/FG, (B) Power spectra of empty room data.

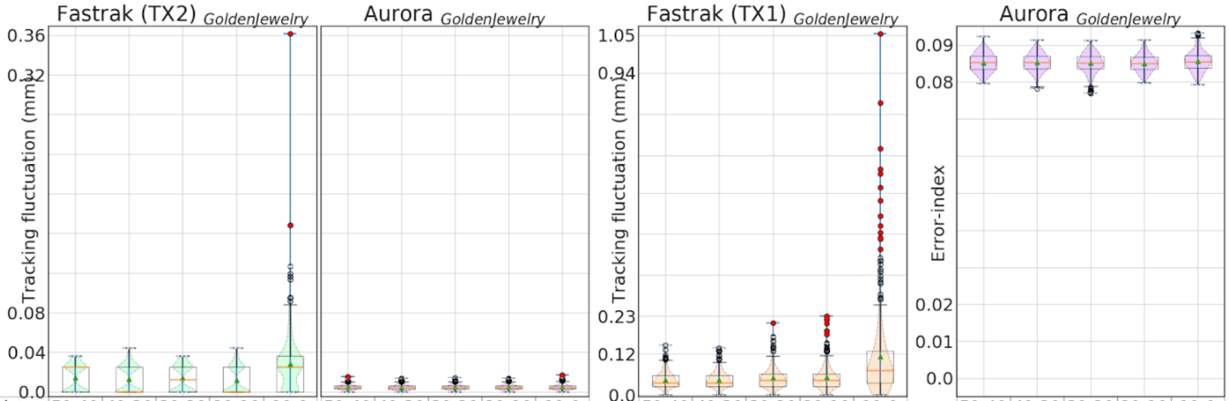


| $Dist_{T-R}$ | 50-40 | 40-30 | 30-20 | 20-10 | 10-0 |
|---|---|---|---|---|---|
| $Mean_*$ | 0.01 | 0.01 | 0.01 | 0.01 | 0.03 |
| $IQR$ | 0.03 | 0.03 | 0.03 | 0.03 | 0.04 |
| $Out.\%$ | 0.0 | 0.0 | 0.0 | 0.0 | 3.04 |
| $Ext.\%$ | 0.0 | 0.0 | 0.0 | 0.0 | 0.65 |

| $Dist_{T-R}$ | 50-40 | 40-30 | 30-20 | 20-10 | 10-0 |
|---|---|---|---|---|---|
| $Mean_*$ | 0.0 | 0.0 | 0.0 | 0.0 | 0.0 |
| $IQR$ | 0.0 | 0.0 | 0.0 | 0.0 | 0.0 |
| $Out.\%$ | 2.67 | 1.83 | 2.17 | 2.67 | 3.5 |
| $Ext.\%$ | 0.33 | 0.0 | 0.0 | 0.0 | 0.33 |

| $Dist_{T-R}$ | 50-40 | 40-30 | 30-20 | 20-10 | 10-0 |
|---|---|---|---|---|---|
| $Mean_*$ | 0.04 | 0.04 | 0.05 | 0.05 | 0.09 |
| $IQR$ | 0.03 | 0.03 | 0.04 | 0.04 | 0.09 |
| $Out.\%$ | 3.04 | 3.34 | 4.26 | 6.08 | 8.51 |
| $Ext.\%$ | 0.0 | 0.0 | 0.3 | 2.43 | 3.95 |

| $Dist_{T-R}$ | 50-40 | 40-30 | 30-20 | 20-10 | 10-0 |
|---|---|---|---|---|---|
| $Mean_*$ | 0.09 | 0.09 | 0.09 | 0.09 | 0.09 |
| $IQR$ | 0.0 | 0.0 | 0.0 | 0.0 | 0.0 |
| $Out.\%$ | 0.0 | 0.0 | 0.0 | 0.0 | 0.83 |
| $Ext.\%$ | 0.0 | 0.0 | 0.0 | 0.0 | 0.0 |

(A)

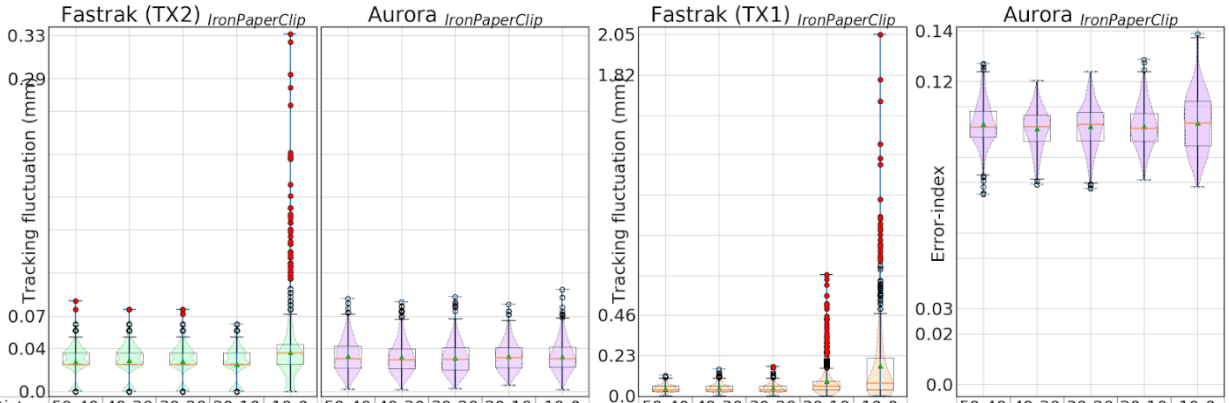


| $Dist_{T-R}$ | 50-40 | 40-30 | 30-20 | 20-10 | 10-0 |
|---|---|---|---|---|---|
| $Mean_*$ | 0.03 | 0.03 | 0.03 | 0.03 | 0.03 |
| $IQR$ | 0.01 | 0.01 | 0.01 | 0.01 | 0.02 |
| $Out.\%$ | 4.12 | 5.9 | 5.09 | 3.72 | 7.92 |
| $Ext.\%$ | 0.16 | 0.16 | 0.32 | 0.0 | 4.45 |

| $Dist_{T-R}$ | 50-40 | 40-30 | 30-20 | 20-10 | 10-0 |
|---|---|---|---|---|---|
| $Mean_*$ | 0.03 | 0.03 | 0.03 | 0.03 | 0.03 |
| $IQR$ | 0.02 | 0.02 | 0.02 | 0.02 | 0.02 |
| $Out.\%$ | 0.83 | 1.67 | 1.17 | 0.83 | 1.33 |
| $Ext.\%$ | 0.0 | 0.0 | 0.0 | 0.0 | 0.0 |

| $Dist_{T-R}$ | 50-40 | 40-30 | 30-20 | 20-10 | 10-0 |
|---|---|---|---|---|---|
| $Mean_*$ | 0.04 | 0.04 | 0.04 | 0.06 | 0.14 |
| $IQR$ | 0.03 | 0.03 | 0.03 | 0.05 | 0.18 |
| $Out.\%$ | 1.03 | 2.23 | 2.41 | 12.03 | 8.25 |
| $Ext.\%$ | 0.0 | 0.0 | 0.34 | 7.22 | 3.78 |

| $Dist_{T-R}$ | 50-40 | 40-30 | 30-20 | 20-10 | 10-0 |
|---|---|---|---|---|---|
| $Mean_*$ | 0.1 | 0.1 | 0.1 | 0.1 | 0.1 |
| $IQR$ | 0.01 | 0.01 | 0.01 | 0.01 | 0.02 |
| $Out.\%$ | 0.83 | 0.0 | 0.0 | 0.5 | 0.17 |
| $Ext.\%$ | 0.0 | 0.0 | 0.0 | 0.0 | 0.0 |

(B)

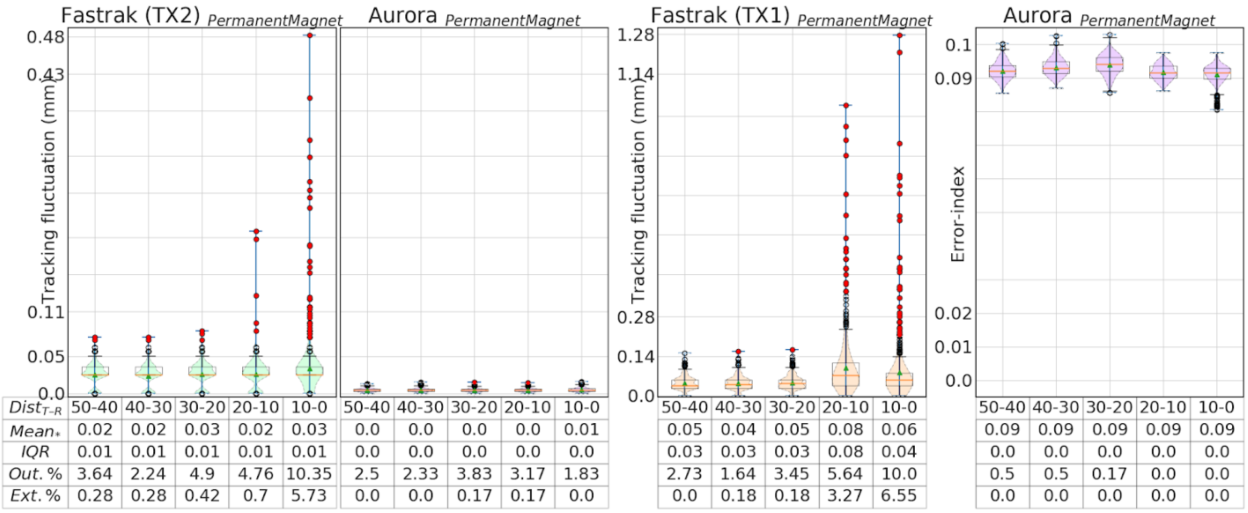


| $Dist_{T-R}$ | 50-40 | 40-30 | 30-20 | 20-10 | 10-0 |
|---|---|---|---|---|---|
| $Mean_*$ | 0.02 | 0.02 | 0.03 | 0.02 | 0.03 |
| $IQR$ | 0.01 | 0.01 | 0.01 | 0.01 | 0.01 |
| $Out.\%$ | 3.64 | 2.24 | 4.9 | 4.76 | 10.35 |
| $Ext.\%$ | 0.28 | 0.28 | 0.42 | 0.7 | 5.73 |

| $Dist_{T-R}$ | 50-40 | 40-30 | 30-20 | 20-10 | 10-0 |
|---|---|---|---|---|---|
| $Mean_*$ | 0.0 | 0.0 | 0.0 | 0.0 | 0.01 |
| $IQR$ | 0.0 | 0.0 | 0.0 | 0.0 | 0.0 |
| $Out.\%$ | 2.5 | 2.33 | 3.83 | 3.17 | 1.83 |
| $Ext.\%$ | 0.0 | 0.0 | 0.17 | 0.17 | 0.0 |

| $Dist_{T-R}$ | 50-40 | 40-30 | 30-20 | 20-10 | 10-0 |
|---|---|---|---|---|---|
| $Mean_*$ | 0.05 | 0.04 | 0.05 | 0.08 | 0.06 |
| $IQR$ | 0.03 | 0.03 | 0.03 | 0.08 | 0.04 |
| $Out.\%$ | 2.73 | 1.64 | 3.45 | 5.64 | 10.0 |
| $Ext.\%$ | 0.0 | 0.18 | 0.18 | 3.27 | 6.55 |

| $Dist_{T-R}$ | 50-40 | 40-30 | 30-20 | 20-10 | 10-0 |
|---|---|---|---|---|---|
| $Mean_*$ | 0.09 | 0.09 | 0.09 | 0.09 | 0.09 |
| $IQR$ | 0.0 | 0.0 | 0.0 | 0.0 | 0.0 |
| $Out.\%$ | 0.5 | 0.5 | 0.17 | 0.0 | 0.0 |
| $Ext.\%$ | 0.0 | 0.0 | 0.0 | 0.0 | 0.0 |

(C)

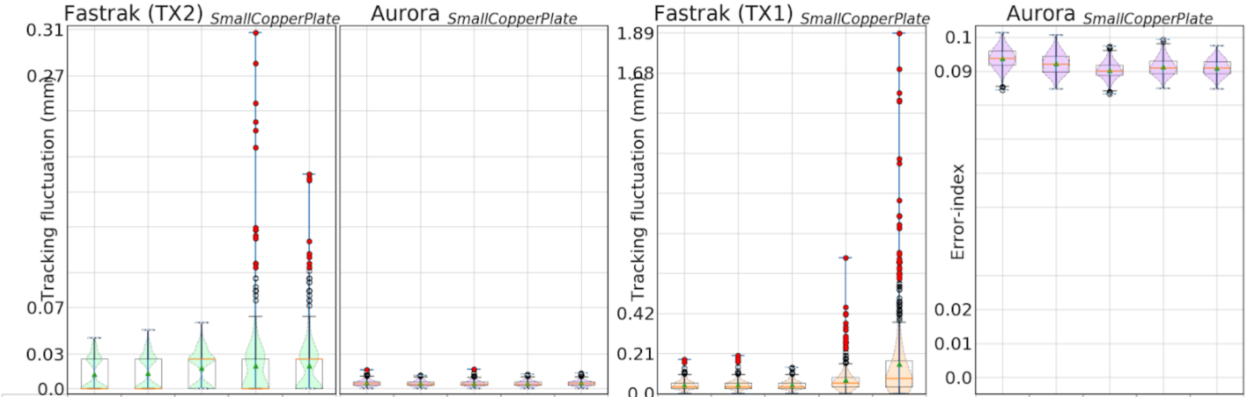


| $Dist_{T-R}$ | 50-40 | 40-30 | 30-20 | 20-10 | 10-0 |
|---|---|---|---|---|---|
| $Mean_*$ | 0.01 | 0.01 | 0.02 | 0.02 | 0.02 |
| IQR | 0.03 | 0.03 | 0.03 | 0.03 | 0.03 |
| Out.% | 0.0 | 0.0 | 0.0 | 4.19 | 4.01 |
| Ext.% | 0.0 | 0.0 | 0.0 | 2.44 | 1.4 |

| $Dist_{T-R}$ | 50-40 | 40-30 | 30-20 | 20-10 | 10-0 |
|---|---|---|---|---|---|
| $Mean_*$ | 0.0 | 0.0 | 0.0 | 0.0 | 0.0 |
| IQR | 0.0 | 0.0 | 0.0 | 0.0 | 0.0 |
| Out.% | 2.5 | 1.33 | 1.83 | 1.0 | 1.83 |
| Ext.% | 0.17 | 0.0 | 0.17 | 0.0 | 0.0 |

| $Dist_{T-R}$ | 50-40 | 40-30 | 30-20 | 20-10 | 10-0 |
|---|---|---|---|---|---|
| $Mean_*$ | 0.04 | 0.04 | 0.04 | 0.06 | 0.11 |
| IQR | 0.03 | 0.03 | 0.03 | 0.05 | 0.14 |
| Out.% | 1.85 | 2.96 | 1.67 | 6.48 | 9.07 |
| Ext.% | 0.37 | 0.74 | 0.0 | 3.15 | 5.0 |

| $Dist_{T-R}$ | 50-40 | 40-30 | 30-20 | 20-10 | 10-0 |
|---|---|---|---|---|---|
| $Mean_*$ | 0.09 | 0.09 | 0.09 | 0.09 | 0.09 |
| IQR | 0.0 | 0.0 | 0.0 | 0.0 | 0.0 |
| Out.% | 0.0 | 0.0 | 0.67 | 0.33 | 0.0 |
| Ext.% | 0.0 | 0.0 | 0.0 | 0.0 | 0.0 |

(D)

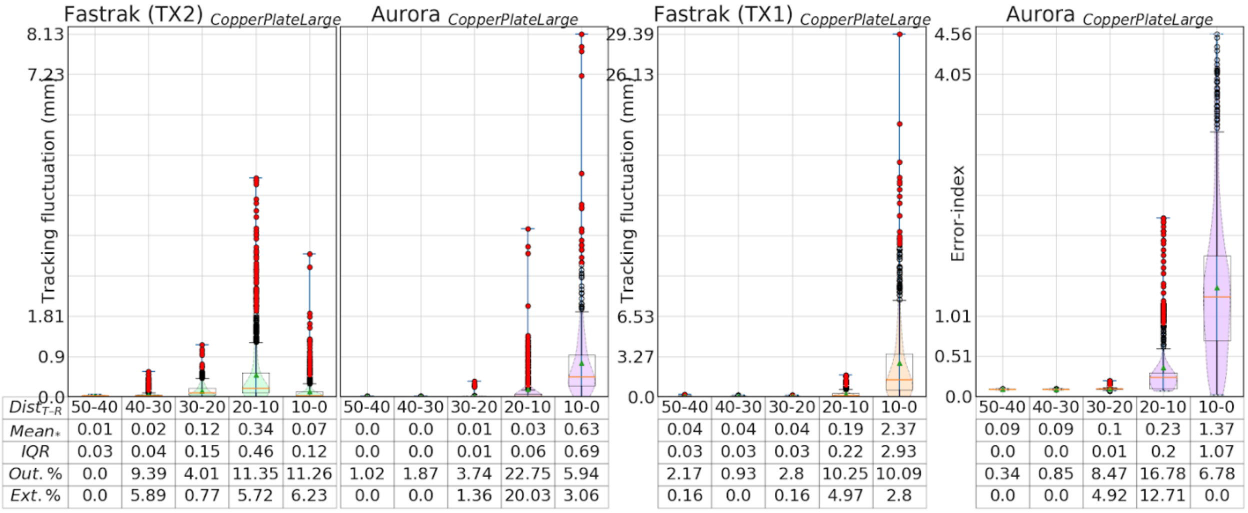


| $Dist_{T-R}$ | 50-40 | 40-30 | 30-20 | 20-10 | 10-0 |
|---|---|---|---|---|---|
| $Mean_*$ | 0.01 | 0.02 | 0.12 | 0.34 | 0.07 |
| IQR | 0.03 | 0.04 | 0.15 | 0.46 | 0.12 |
| Out.% | 0.0 | 9.39 | 4.01 | 11.35 | 11.26 |
| Ext.% | 0.0 | 5.89 | 0.77 | 5.72 | 6.23 |

| $Dist_{T-R}$ | 50-40 | 40-30 | 30-20 | 20-10 | 10-0 |
|---|---|---|---|---|---|
| $Mean_*$ | 0.0 | 0.0 | 0.01 | 0.03 | 0.63 |
| IQR | 0.0 | 0.0 | 0.01 | 0.06 | 0.69 |
| Out.% | 1.02 | 1.87 | 3.74 | 22.75 | 5.94 |
| Ext.% | 0.0 | 0.0 | 1.36 | 20.03 | 3.06 |

| $Dist_{T-R}$ | 50-40 | 40-30 | 30-20 | 20-10 | 10-0 |
|---|---|---|---|---|---|
| $Mean_*$ | 0.04 | 0.04 | 0.04 | 0.19 | 2.37 |
| IQR | 0.03 | 0.03 | 0.03 | 0.22 | 2.93 |
| Out.% | 2.17 | 0.93 | 2.8 | 10.25 | 10.09 |
| Ext.% | 0.16 | 0.0 | 0.16 | 4.97 | 2.8 |

| $Dist_{T-R}$ | 50-40 | 40-30 | 30-20 | 20-10 | 10-0 |
|---|---|---|---|---|---|
| $Mean_*$ | 0.09 | 0.09 | 0.1 | 0.23 | 1.37 |
| IQR | 0.0 | 0.0 | 0.01 | 0.2 | 1.07 |
| Out.% | 0.34 | 0.85 | 8.47 | 16.78 | 6.78 |
| Ext.% | 0.0 | 0.0 | 4.92 | 12.71 | 0.0 |

(E)

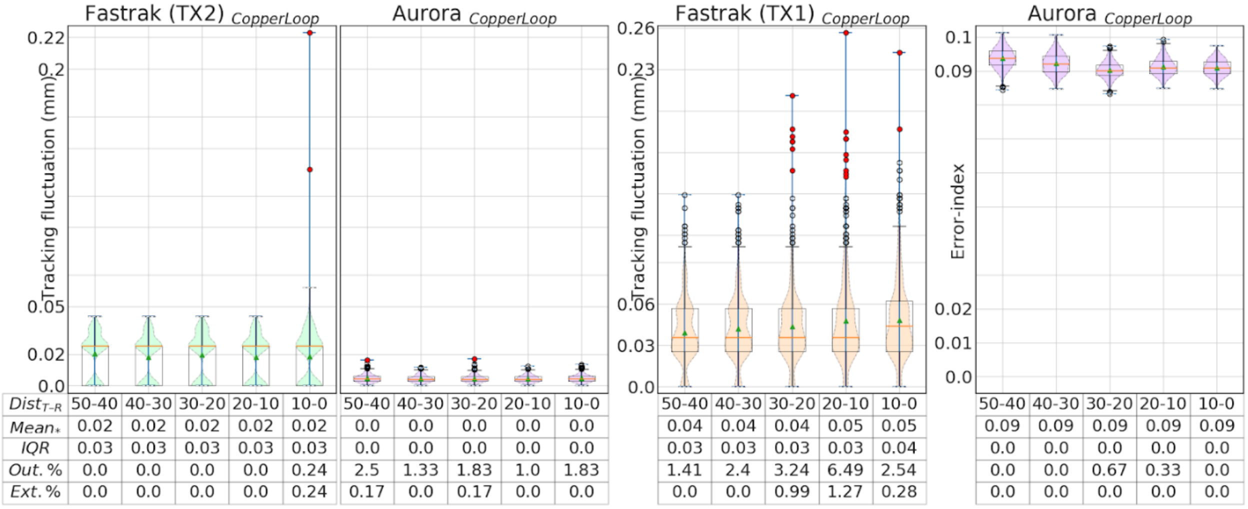


| $Dist_{T-R}$ | 50-40 | 40-30 | 30-20 | 20-10 | 10-0 |
|---|---|---|---|---|---|
| $Mean_*$ | 0.02 | 0.02 | 0.02 | 0.02 | 0.02 |
| IQR | 0.03 | 0.03 | 0.03 | 0.03 | 0.03 |
| Out.% | 0.0 | 0.0 | 0.0 | 0.0 | 0.24 |
| Ext.% | 0.0 | 0.0 | 0.0 | 0.0 | 0.24 |

| $Dist_{T-R}$ | 50-40 | 40-30 | 30-20 | 20-10 | 10-0 |
|---|---|---|---|---|---|
| $Mean_*$ | 0.0 | 0.0 | 0.0 | 0.0 | 0.0 |
| IQR | 0.0 | 0.0 | 0.0 | 0.0 | 0.0 |
| Out.% | 2.5 | 1.33 | 1.83 | 1.0 | 1.83 |
| Ext.% | 0.17 | 0.0 | 0.17 | 0.0 | 0.0 |

| $Dist_{T-R}$ | 50-40 | 40-30 | 30-20 | 20-10 | 10-0 |
|---|---|---|---|---|---|
| $Mean_*$ | 0.04 | 0.04 | 0.04 | 0.05 | 0.05 |
| IQR | 0.03 | 0.03 | 0.03 | 0.03 | 0.04 |
| Out.% | 1.41 | 2.4 | 3.24 | 6.49 | 2.54 |
| Ext.% | 0.0 | 0.0 | 0.99 | 1.27 | 0.28 |

| $Dist_{T-R}$ | 50-40 | 40-30 | 30-20 | 20-10 | 10-0 |
|---|---|---|---|---|---|
| $Mean_*$ | 0.09 | 0.09 | 0.09 | 0.09 | 0.09 |
| IQR | 0.0 | 0.0 | 0.0 | 0.0 | 0.0 |
| Out.% | 0.0 | 0.0 | 0.67 | 0.33 | 0.0 |
| Ext.% | 0.0 | 0.0 | 0.0 | 0.0 | 0.0 |

(F)

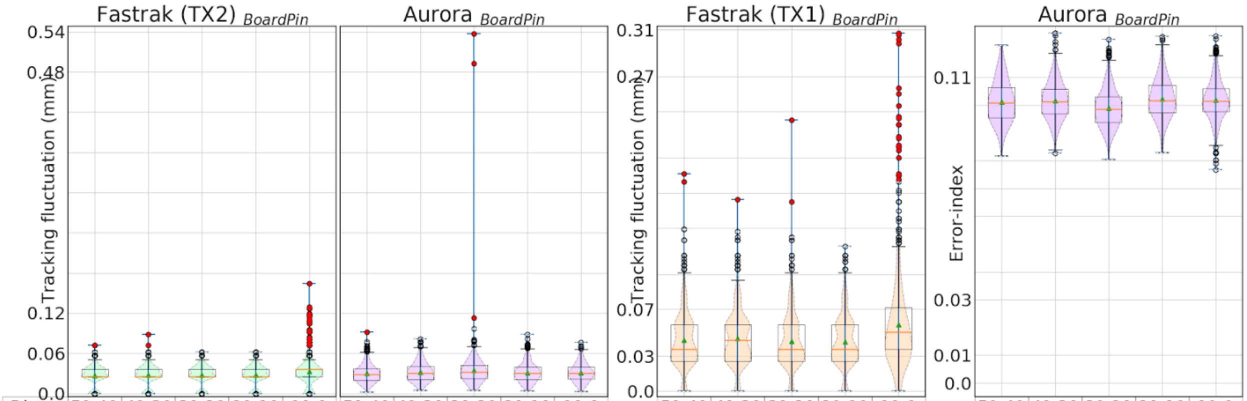


| $Dist_{T-R}$ | 50-40 | 40-30 | 30-20 | 20-10 | 10-0 | 50-40 | 40-30 | 30-20 | 20-10 | 10-0 | 50-40 | 40-30 | 30-20 | 20-10 | 10-0 | 50-40 | 40-30 | 30-20 | 20-10 | 10-0 |
|---|---|---|---|---|---|---|---|---|---|---|---|---|---|---|---|---|---|---|---|---|
| $Mean_*$ | 0.03 | 0.03 | 0.03 | 0.03 | 0.03 | 0.03 | 0.03 | 0.03 | 0.03 | 0.03 | 0.04 | 0.04 | 0.04 | 0.04 | 0.05 | 0.1 | 0.1 | 0.1 | 0.1 | 0.1 |
| IQR | 0.01 | 0.01 | 0.01 | 0.01 | 0.01 | 0.02 | 0.02 | 0.02 | 0.02 | 0.02 | 0.03 | 0.03 | 0.03 | 0.03 | 0.04 | 0.01 | 0.01 | 0.01 | 0.01 | 0.01 |
| Out. % | 4.44 | 5.39 | 3.96 | 5.39 | 14.42 | 2.33 | 1.5 | 1.67 | 2.0 | 0.67 | 2.96 | 2.56 | 2.29 | 1.35 | 6.59 | 0.0 | 1.16 | 1.66 | 0.67 | 2.83 |
| Ext. % | 0.32 | 0.32 | 0.0 | 0.0 | 5.86 | 0.17 | 0.0 | 0.5 | 0.0 | 0.0 | 0.27 | 0.13 | 0.27 | 0.0 | 2.96 | 0.0 | 0.0 | 0.0 | 0.0 | 0.0 |

(G)

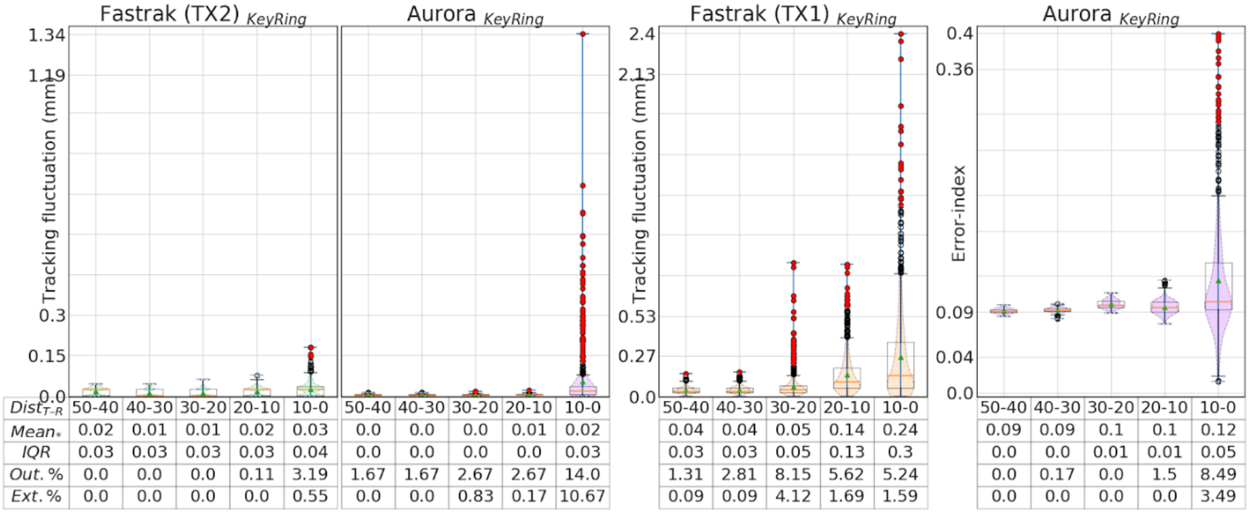


| $Dist_{T-R}$ | 50-40 | 40-30 | 30-20 | 20-10 | 10-0 | 50-40 | 40-30 | 30-20 | 20-10 | 10-0 | 50-40 | 40-30 | 30-20 | 20-10 | 10-0 | 50-40 | 40-30 | 30-20 | 20-10 | 10-0 |
|---|---|---|---|---|---|---|---|---|---|---|---|---|---|---|---|---|---|---|---|---|
| $Mean_*$ | 0.02 | 0.01 | 0.01 | 0.02 | 0.03 | 0.0 | 0.0 | 0.0 | 0.01 | 0.02 | 0.04 | 0.04 | 0.05 | 0.14 | 0.24 | 0.09 | 0.09 | 0.1 | 0.1 | 0.12 |
| IQR | 0.03 | 0.03 | 0.03 | 0.03 | 0.04 | 0.0 | 0.0 | 0.0 | 0.0 | 0.03 | 0.03 | 0.03 | 0.05 | 0.13 | 0.3 | 0.0 | 0.0 | 0.01 | 0.01 | 0.05 |
| Out. % | 0.0 | 0.0 | 0.0 | 0.11 | 3.19 | 1.67 | 1.67 | 2.67 | 2.67 | 14.0 | 1.31 | 2.81 | 8.15 | 5.62 | 5.24 | 0.0 | 0.17 | 0.0 | 1.5 | 8.49 |
| Ext. % | 0.0 | 0.0 | 0.0 | 0.0 | 0.55 | 0.0 | 0.0 | 0.83 | 0.17 | 10.67 | 0.09 | 0.09 | 4.12 | 1.69 | 1.59 | 0.0 | 0.0 | 0.0 | 0.0 | 3.49 |

(H)

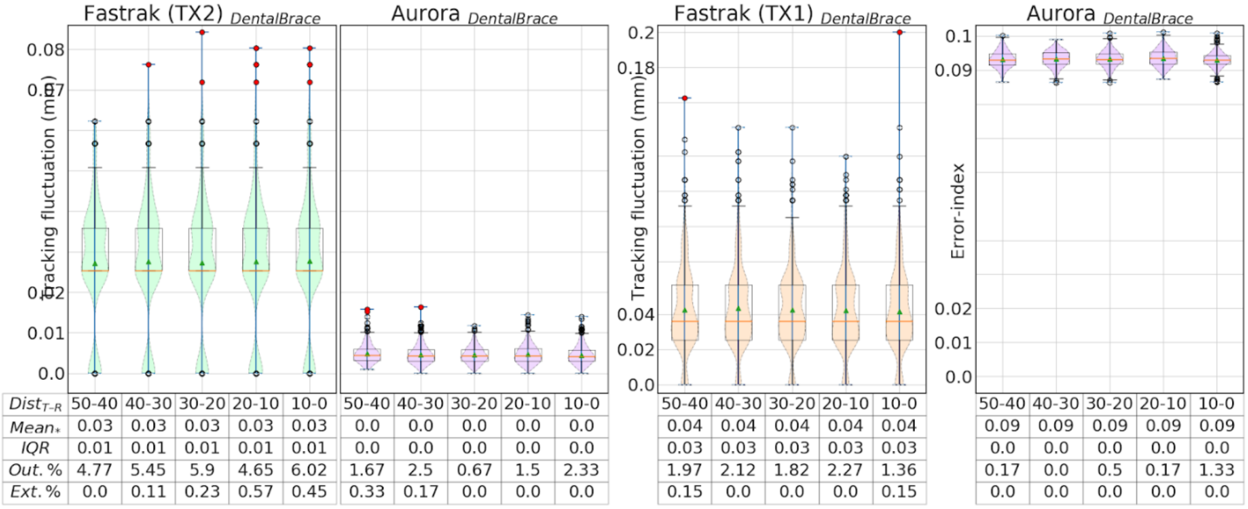


| $Dist_{T-R}$ | 50-40 | 40-30 | 30-20 | 20-10 | 10-0 | 50-40 | 40-30 | 30-20 | 20-10 | 10-0 | 50-40 | 40-30 | 30-20 | 20-10 | 10-0 | 50-40 | 40-30 | 30-20 | 20-10 | 10-0 |
|---|---|---|---|---|---|---|---|---|---|---|---|---|---|---|---|---|---|---|---|---|
| $Mean_*$ | 0.03 | 0.03 | 0.03 | 0.03 | 0.03 | 0.0 | 0.0 | 0.0 | 0.0 | 0.0 | 0.04 | 0.04 | 0.04 | 0.04 | 0.04 | 0.09 | 0.09 | 0.09 | 0.09 | 0.09 |
| IQR | 0.01 | 0.01 | 0.01 | 0.01 | 0.01 | 0.0 | 0.0 | 0.0 | 0.0 | 0.0 | 0.03 | 0.03 | 0.03 | 0.03 | 0.03 | 0.0 | 0.0 | 0.0 | 0.0 | 0.0 |
| Out. % | 4.77 | 5.45 | 5.9 | 4.65 | 6.02 | 1.67 | 2.5 | 0.67 | 1.5 | 2.33 | 1.97 | 2.12 | 1.82 | 2.27 | 1.36 | 0.17 | 0.0 | 0.5 | 0.17 | 1.33 |
| Ext. % | 0.0 | 0.11 | 0.23 | 0.57 | 0.45 | 0.33 | 0.17 | 0.0 | 0.0 | 0.0 | 0.15 | 0.0 | 0.0 | 0.0 | 0.15 | 0.0 | 0.0 | 0.0 | 0.0 | 0.0 |

(I)

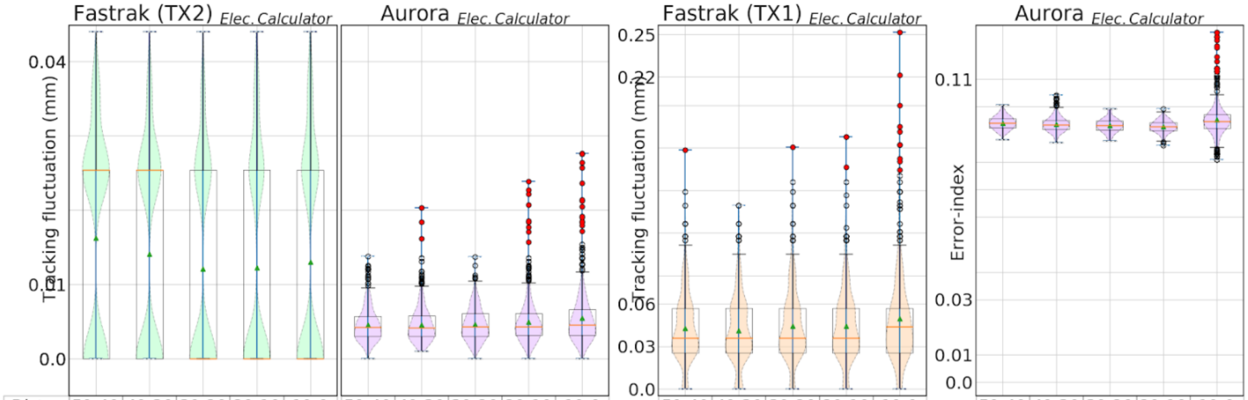


| $Dist_{T-R}$ | Fastrak (TX2) 50-40 | 40-30 | 30-20 | 20-10 | 10-0 | Aurora 50-40 | 40-30 | 30-20 | 20-10 | 10-0 | Fastrak (TX1) 50-40 | 40-30 | 30-20 | 20-10 | 10-0 | Aurora 50-40 | 40-30 | 30-20 | 20-10 | 10-0 |
|---|---|---|---|---|---|---|---|---|---|---|---|---|---|---|---|---|---|---|---|---|
| $Mean_*$ | 0.02 | 0.01 | 0.01 | 0.01 | 0.01 | 0.0 | 0.0 | 0.0 | 0.0 | 0.01 | 0.04 | 0.04 | 0.04 | 0.04 | 0.05 | 0.09 | 0.09 | 0.09 | 0.09 | 0.09 |
| IQR | 0.03 | 0.03 | 0.03 | 0.03 | 0.03 | 0.0 | 0.0 | 0.0 | 0.0 | 0.0 | 0.03 | 0.03 | 0.03 | 0.03 | 0.03 | 0.0 | 0.0 | 0.0 | 0.0 | 0.0 |
| Out. % | 0.0 | 0.0 | 0.0 | 0.0 | 0.0 | 2.67 | 3.33 | 1.0 | 4.33 | 4.67 | 2.03 | 2.26 | 2.93 | 3.61 | 6.55 | 0.0 | 2.17 | 0.0 | 0.17 | 4.67 |
| Ext. % | 0.0 | 0.0 | 0.0 | 0.0 | 0.0 | 0.0 | 0.5 | 0.0 | 1.5 | 2.33 | 0.23 | 0.0 | 0.23 | 0.68 | 2.26 | 0.0 | 0.0 | 0.0 | 0.0 | 2.5 |

(J)

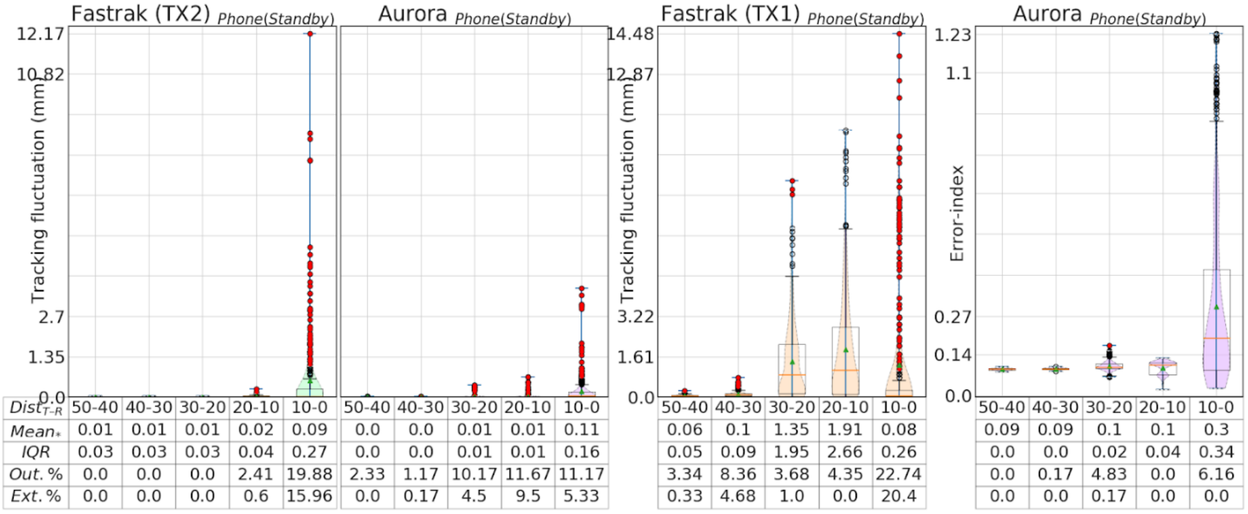


| $Dist_{T-R}$ | Fastrak (TX2) 50-40 | 40-30 | 30-20 | 20-10 | 10-0 | Aurora 50-40 | 40-30 | 30-20 | 20-10 | 10-0 | Fastrak (TX1) 50-40 | 40-30 | 30-20 | 20-10 | 10-0 | Aurora 50-40 | 40-30 | 30-20 | 20-10 | 10-0 |
|---|---|---|---|---|---|---|---|---|---|---|---|---|---|---|---|---|---|---|---|---|
| $Mean_*$ | 0.01 | 0.01 | 0.01 | 0.02 | 0.09 | 0.0 | 0.0 | 0.01 | 0.01 | 0.11 | 0.06 | 0.1 | 1.35 | 1.91 | 0.08 | 0.09 | 0.09 | 0.1 | 0.1 | 0.3 |
| IQR | 0.03 | 0.03 | 0.03 | 0.04 | 0.27 | 0.0 | 0.0 | 0.01 | 0.01 | 0.16 | 0.05 | 0.09 | 1.95 | 2.66 | 0.26 | 0.0 | 0.0 | 0.02 | 0.04 | 0.34 |
| Out. % | 0.0 | 0.0 | 0.0 | 2.41 | 19.88 | 2.33 | 1.17 | 10.17 | 11.67 | 11.17 | 3.34 | 8.36 | 3.68 | 4.35 | 22.74 | 0.0 | 0.17 | 4.83 | 0.0 | 6.16 |
| Ext. % | 0.0 | 0.0 | 0.0 | 0.6 | 15.96 | 0.0 | 0.17 | 4.5 | 9.5 | 5.33 | 0.33 | 4.68 | 1.0 | 0.0 | 20.4 | 0.0 | 0.0 | 0.17 | 0.0 | 0.0 |

(K)

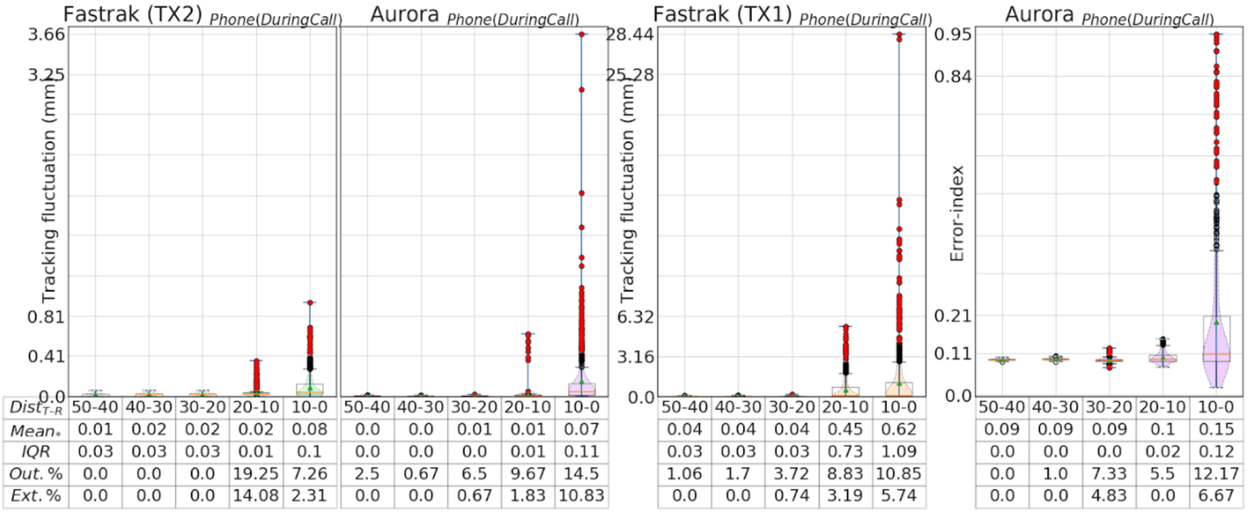


| $Dist_{T-R}$ | Fastrak (TX2) 50-40 | 40-30 | 30-20 | 20-10 | 10-0 | Aurora 50-40 | 40-30 | 30-20 | 20-10 | 10-0 | Fastrak (TX1) 50-40 | 40-30 | 30-20 | 20-10 | 10-0 | Aurora 50-40 | 40-30 | 30-20 | 20-10 | 10-0 |
|---|---|---|---|---|---|---|---|---|---|---|---|---|---|---|---|---|---|---|---|---|
| $Mean_*$ | 0.01 | 0.02 | 0.02 | 0.02 | 0.08 | 0.0 | 0.0 | 0.01 | 0.01 | 0.07 | 0.04 | 0.04 | 0.04 | 0.45 | 0.62 | 0.09 | 0.09 | 0.09 | 0.1 | 0.15 |
| IQR | 0.03 | 0.03 | 0.03 | 0.01 | 0.1 | 0.0 | 0.0 | 0.0 | 0.01 | 0.11 | 0.03 | 0.03 | 0.03 | 0.73 | 1.09 | 0.0 | 0.0 | 0.0 | 0.02 | 0.12 |
| Out. % | 0.0 | 0.0 | 0.0 | 19.25 | 7.26 | 2.5 | 0.67 | 6.5 | 9.67 | 14.5 | 1.06 | 1.7 | 3.72 | 8.83 | 10.85 | 0.0 | 1.0 | 7.33 | 5.5 | 12.17 |
| Ext. % | 0.0 | 0.0 | 0.0 | 14.08 | 2.31 | 0.0 | 0.0 | 0.67 | 1.83 | 10.83 | 0.0 | 0.0 | 0.74 | 3.19 | 5.74 | 0.0 | 0.0 | 4.83 | 0.0 | 6.67 |

(L)

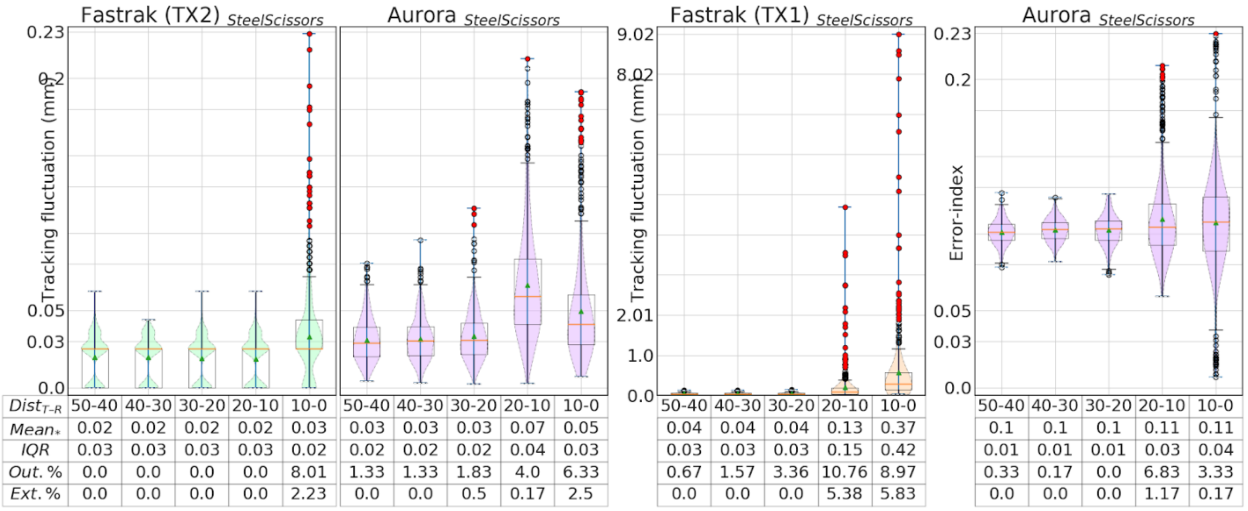


| $Dist_{T-R}$ | 50-40 | 40-30 | 30-20 | 20-10 | 10-0 | 50-40 | 40-30 | 30-20 | 20-10 | 10-0 |
|---|---|---|---|---|---|---|---|---|---|---|
| $Mean_*$ | 0.02 | 0.02 | 0.02 | 0.02 | 0.03 | 0.03 | 0.03 | 0.03 | 0.07 | 0.05 |
| IQR | 0.03 | 0.03 | 0.03 | 0.03 | 0.02 | 0.02 | 0.02 | 0.02 | 0.04 | 0.03 |
| Out.% | 0.0 | 0.0 | 0.0 | 0.0 | 8.01 | 1.33 | 1.33 | 1.83 | 4.0 | 6.33 |
| Ext.% | 0.0 | 0.0 | 0.0 | 0.0 | 2.23 | 0.0 | 0.0 | 0.5 | 0.17 | 2.5 |

| 50-40 | 40-30 | 30-20 | 20-10 | 10-0 |
|---|---|---|---|---|
| 0.04 | 0.04 | 0.04 | 0.13 | 0.37 |
| 0.03 | 0.03 | 0.03 | 0.15 | 0.42 |
| 0.67 | 1.57 | 3.36 | 10.76 | 8.97 |
| 0.0 | 0.0 | 0.0 | 5.38 | 5.83 |

| 50-40 | 40-30 | 30-20 | 20-10 | 10-0 |
|---|---|---|---|---|
| 0.1 | 0.1 | 0.1 | 0.11 | 0.11 |
| 0.01 | 0.01 | 0.01 | 0.03 | 0.04 |
| 0.33 | 0.17 | 0.0 | 6.83 | 3.33 |
| 0.0 | 0.0 | 0.0 | 1.17 | 0.17 |

(M)

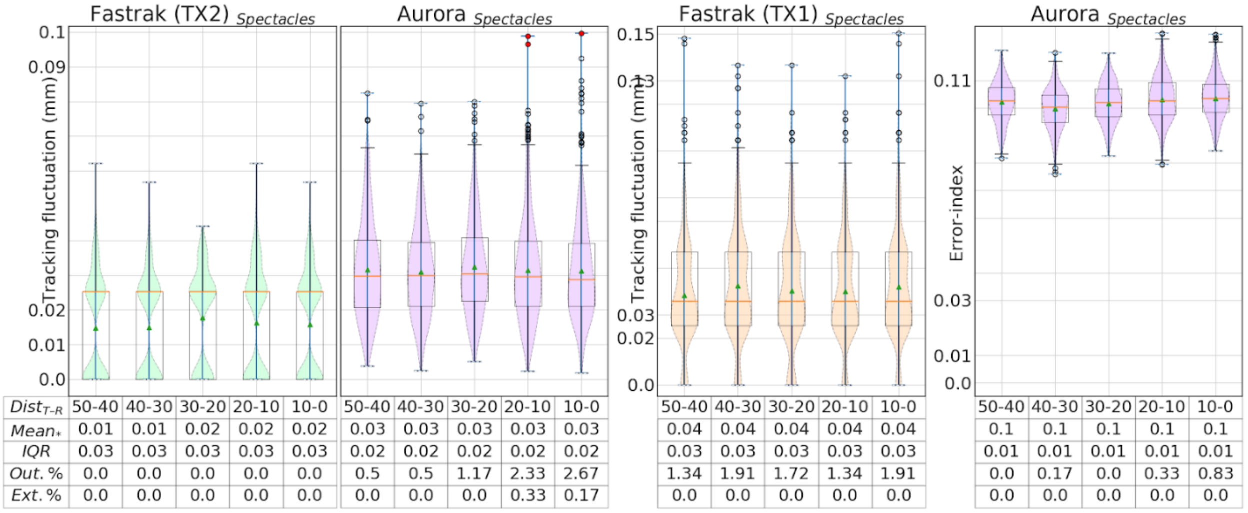


| $Dist_{T-R}$ | 50-40 | 40-30 | 30-20 | 20-10 | 10-0 | 50-40 | 40-30 | 30-20 | 20-10 | 10-0 |
|---|---|---|---|---|---|---|---|---|---|---|
| $Mean_*$ | 0.01 | 0.01 | 0.02 | 0.02 | 0.02 | 0.03 | 0.03 | 0.03 | 0.03 | 0.03 |
| IQR | 0.03 | 0.03 | 0.03 | 0.03 | 0.03 | 0.02 | 0.02 | 0.02 | 0.02 | 0.02 |
| Out.% | 0.0 | 0.0 | 0.0 | 0.0 | 0.0 | 0.5 | 0.5 | 1.17 | 2.33 | 2.67 |
| Ext.% | 0.0 | 0.0 | 0.0 | 0.0 | 0.0 | 0.0 | 0.0 | 0.0 | 0.33 | 0.17 |

| 50-40 | 40-30 | 30-20 | 20-10 | 10-0 |
|---|---|---|---|---|
| 0.04 | 0.04 | 0.04 | 0.04 | 0.04 |
| 0.03 | 0.03 | 0.03 | 0.03 | 0.03 |
| 1.34 | 1.91 | 1.72 | 1.34 | 1.91 |
| 0.0 | 0.0 | 0.0 | 0.0 | 0.0 |

| 50-40 | 40-30 | 30-20 | 20-10 | 10-0 |
|---|---|---|---|---|
| 0.1 | 0.1 | 0.1 | 0.1 | 0.1 |
| 0.01 | 0.01 | 0.01 | 0.01 | 0.01 |
| 0.0 | 0.17 | 0.0 | 0.33 | 0.83 |
| 0.0 | 0.0 | 0.0 | 0.0 | 0.0 |

(N)

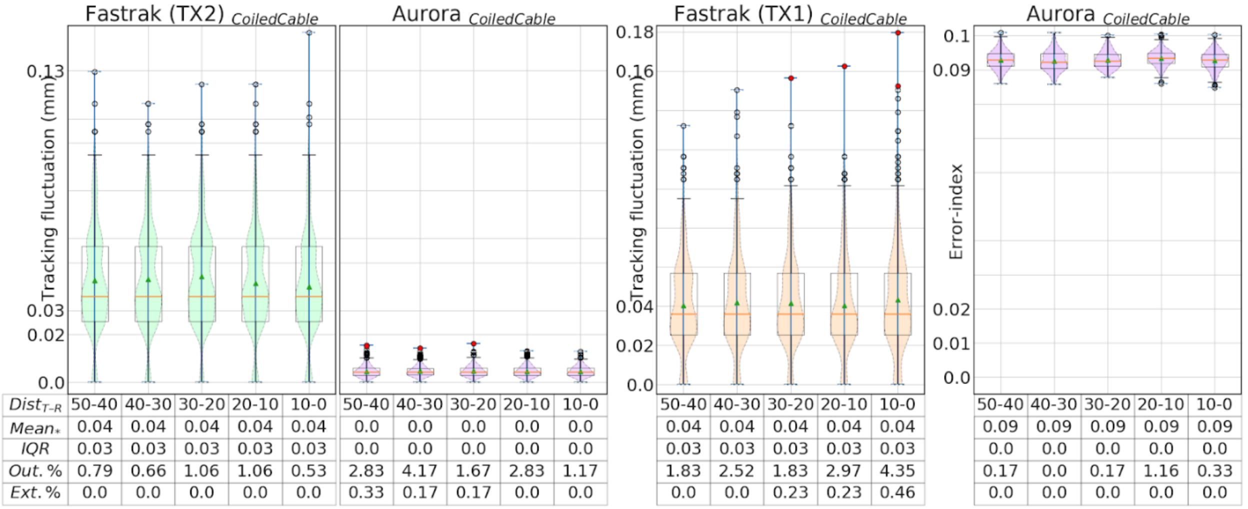


| $Dist_{T-R}$ | 50-40 | 40-30 | 30-20 | 20-10 | 10-0 | 50-40 | 40-30 | 30-20 | 20-10 | 10-0 |
|---|---|---|---|---|---|---|---|---|---|---|
| $Mean_*$ | 0.04 | 0.04 | 0.04 | 0.04 | 0.04 | 0.0 | 0.0 | 0.0 | 0.0 | 0.0 |
| IQR | 0.03 | 0.03 | 0.03 | 0.03 | 0.03 | 0.0 | 0.0 | 0.0 | 0.0 | 0.0 |
| Out.% | 0.79 | 0.66 | 1.06 | 1.06 | 0.53 | 2.83 | 4.17 | 1.67 | 2.83 | 1.17 |
| Ext.% | 0.0 | 0.0 | 0.0 | 0.0 | 0.0 | 0.33 | 0.17 | 0.17 | 0.0 | 0.0 |

| 50-40 | 40-30 | 30-20 | 20-10 | 10-0 |
|---|---|---|---|---|
| 0.04 | 0.04 | 0.04 | 0.04 | 0.04 |
| 0.03 | 0.03 | 0.03 | 0.03 | 0.03 |
| 1.83 | 2.52 | 1.83 | 2.97 | 4.35 |
| 0.0 | 0.0 | 0.23 | 0.23 | 0.46 |

| 50-40 | 40-30 | 30-20 | 20-10 | 10-0 |
|---|---|---|---|---|
| 0.09 | 0.09 | 0.09 | 0.09 | 0.09 |
| 0.0 | 0.0 | 0.0 | 0.0 | 0.0 |
| 0.17 | 0.0 | 0.17 | 1.16 | 0.33 |
| 0.0 | 0.0 | 0.0 | 0.0 | 0.0 |

(O)

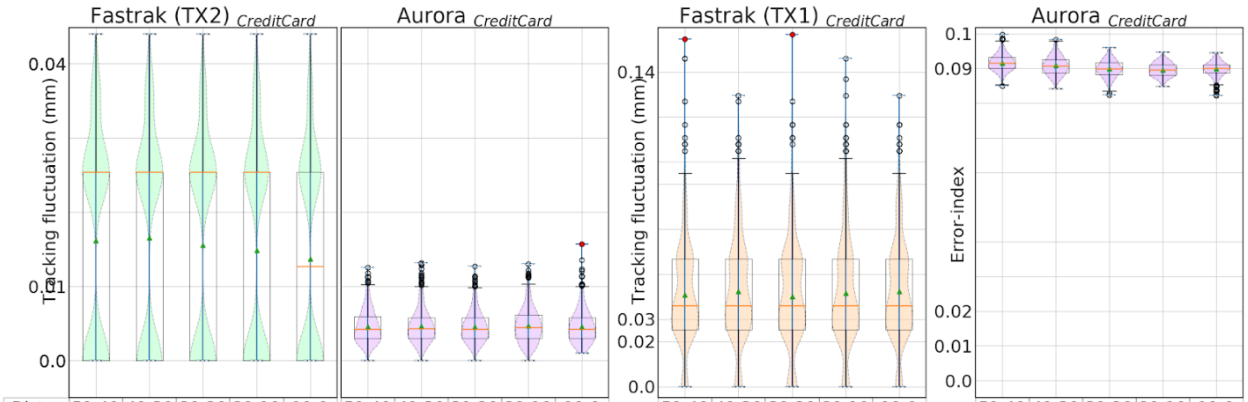


| $Dist_{T-R}$ | 50-40 | 40-30 | 30-20 | 20-10 | 10-0 | 50-40 | 40-30 | 30-20 | 20-10 | 10-0 | 50-40 | 40-30 | 30-20 | 20-10 | 10-0 | 50-40 | 40-30 | 30-20 | 20-10 | 10-0 |
|---|---|---|---|---|---|---|---|---|---|---|---|---|---|---|---|---|---|---|---|---|
| $Mean_*$ | 0.02 | 0.02 | 0.02 | 0.01 | 0.01 | 0.0 | 0.0 | 0.0 | 0.0 | 0.0 | 0.04 | 0.04 | 0.04 | 0.04 | 0.04 | 0.09 | 0.09 | 0.09 | 0.09 | 0.09 |
| $IQR$ | 0.03 | 0.03 | 0.03 | 0.03 | 0.03 | 0.0 | 0.0 | 0.0 | 0.0 | 0.0 | 0.03 | 0.03 | 0.03 | 0.03 | 0.03 | 0.0 | 0.0 | 0.0 | 0.0 | 0.0 |
| $Out.\%$ | 0.0 | 0.0 | 0.0 | 0.0 | 0.0 | 1.33 | 2.5 | 2.0 | 1.67 | 2.33 | 1.58 | 1.76 | 1.23 | 2.82 | 1.58 | 0.83 | 0.17 | 0.0 | 0.0 | 0.0 |
| $Ext.\%$ | 0.0 | 0.0 | 0.0 | 0.0 | 0.0 | 0.0 | 0.0 | 0.0 | 0.0 | 0.17 | 0.18 | 0.0 | 0.18 | 0.0 | 0.0 | 0.0 | 0.0 | 0.0 | 0.0 | 0.0 |

(P)

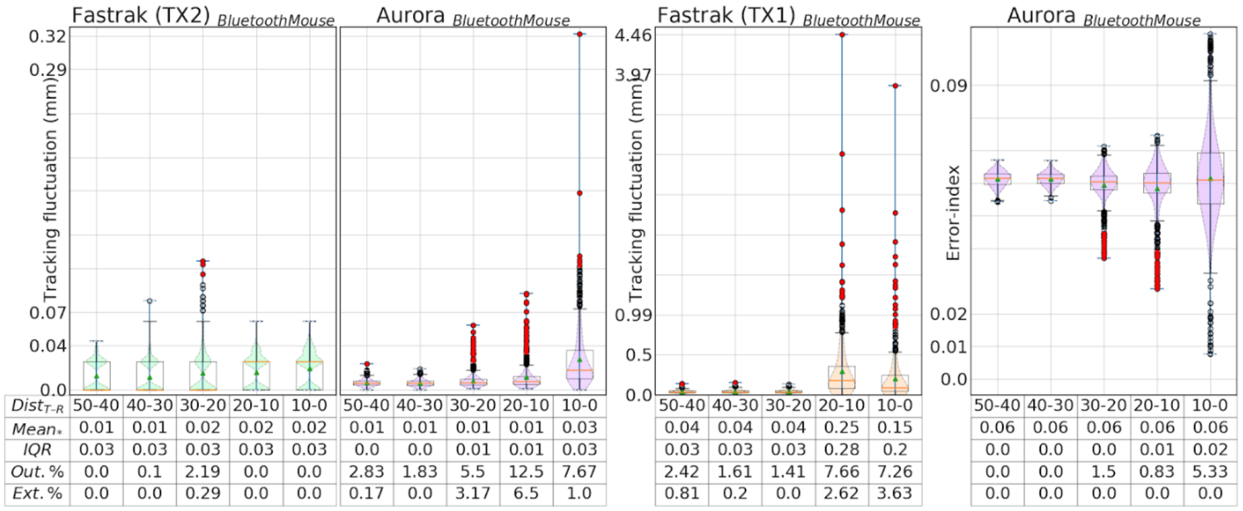


| $Dist_{T-R}$ | 50-40 | 40-30 | 30-20 | 20-10 | 10-0 | 50-40 | 40-30 | 30-20 | 20-10 | 10-0 | 50-40 | 40-30 | 30-20 | 20-10 | 10-0 | 50-40 | 40-30 | 30-20 | 20-10 | 10-0 |
|---|---|---|---|---|---|---|---|---|---|---|---|---|---|---|---|---|---|---|---|---|
| $Mean_*$ | 0.01 | 0.01 | 0.02 | 0.02 | 0.02 | 0.01 | 0.01 | 0.01 | 0.01 | 0.03 | 0.04 | 0.04 | 0.04 | 0.25 | 0.15 | 0.06 | 0.06 | 0.06 | 0.06 | 0.06 |
| $IQR$ | 0.03 | 0.03 | 0.03 | 0.03 | 0.03 | 0.0 | 0.0 | 0.01 | 0.01 | 0.03 | 0.03 | 0.03 | 0.03 | 0.28 | 0.2 | 0.0 | 0.0 | 0.0 | 0.01 | 0.02 |
| $Out.\%$ | 0.0 | 0.1 | 2.19 | 0.0 | 0.0 | 2.83 | 1.83 | 5.5 | 12.5 | 7.67 | 2.42 | 1.61 | 1.41 | 7.66 | 7.26 | 0.0 | 0.0 | 1.5 | 0.83 | 5.33 |
| $Ext.\%$ | 0.0 | 0.0 | 0.29 | 0.0 | 0.0 | 0.17 | 0.0 | 3.17 | 6.5 | 1.0 | 0.81 | 0.2 | 0.0 | 2.62 | 3.63 | 0.0 | 0.0 | 0.0 | 0.0 | 0.0 |

(Q)

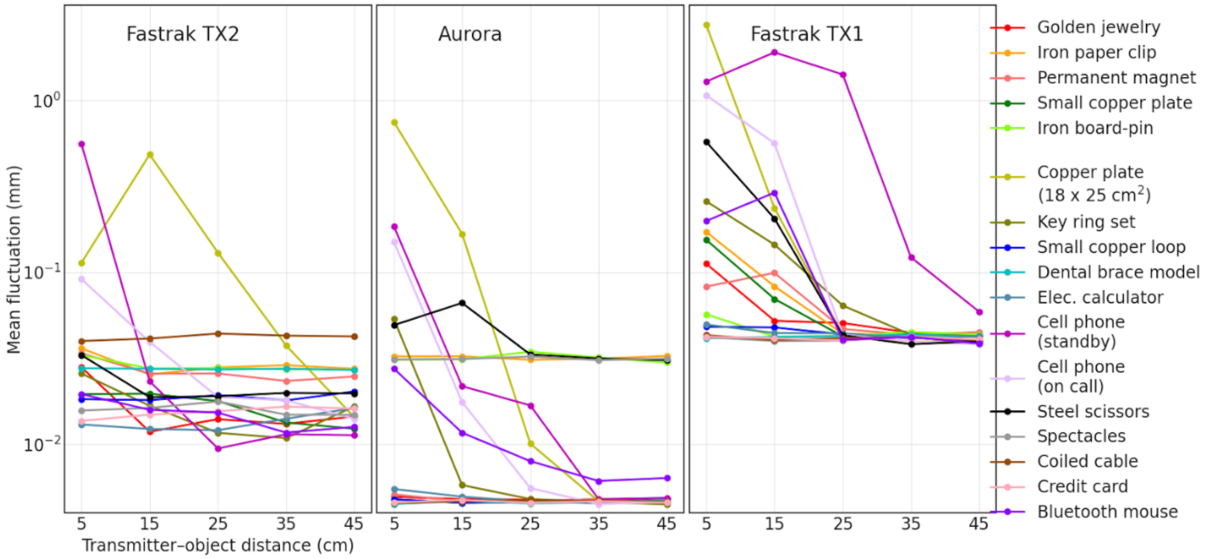


(R)

**Figure S4.** Tracking fluctuation for the systems in the presence of different objects at varying distance from the FG and stylus: A) Jewelry (golden ring, Ø = 2 cm); B) Paper clip (iron, 2 cm); C) Permanent magnet (Ø = 2 cm); D) Copper plate (2 cm x 2 cm); E) Copper plate (18 cm x 25 cm); F) Copper loop (Ø = 4 cm); G) Board pin (iron, 1.5 cm); H) Key ring (iron, 10 cm); I) Dental brace; J) Electronic calculator (switched on); K) Mobile phone (standby); L) Mobile phone (on call); M) Scissors (stainless steel, 15 cm); N) Spectacles (metallic frame); O) Phone charging cable (coiled, Ø = 4 cm); P) Credit card; Q) Bluetooth mouse; and R) Mean fluctuation for all the three systems at five distances

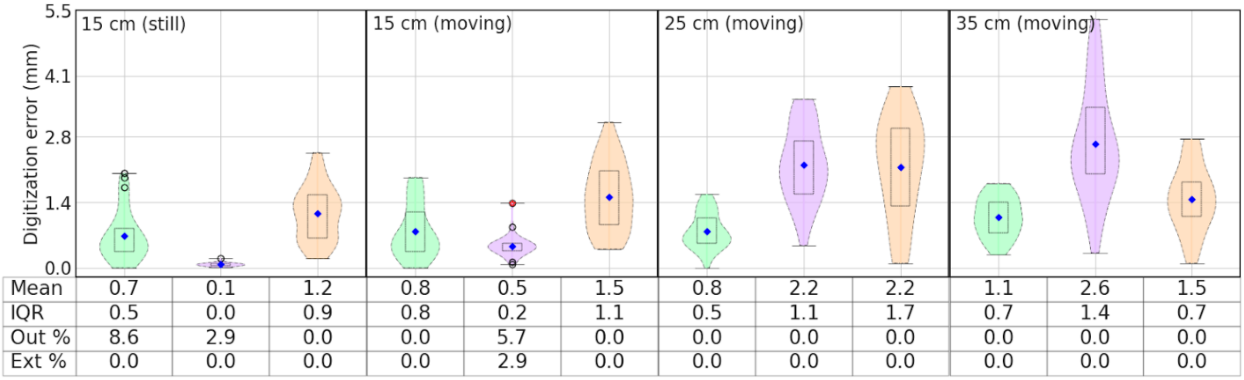


| | | | | | | | | | | | | |
|---|---|---|---|---|---|---|---|---|---|---|---|---|
| Mean | 0.7 | 0.1 | 1.2 | 0.8 | 0.5 | 1.5 | 0.8 | 2.2 | 2.2 | 1.1 | 2.6 | 1.5 |
| IQR | 0.5 | 0.0 | 0.9 | 0.8 | 0.2 | 1.1 | 0.5 | 1.1 | 1.7 | 0.7 | 1.4 | 0.7 |
| Out % | 8.6 | 2.9 | 0.0 | 0.0 | 5.7 | 0.0 | 0.0 | 0.0 | 0.0 | 0.0 | 0.0 | 0.0 |
| Ext % | 0.0 | 0.0 | 0.0 | 0.0 | 2.9 | 0.0 | 0.0 | 0.0 | 0.0 | 0.0 | 0.0 | 0.0 |

(A)

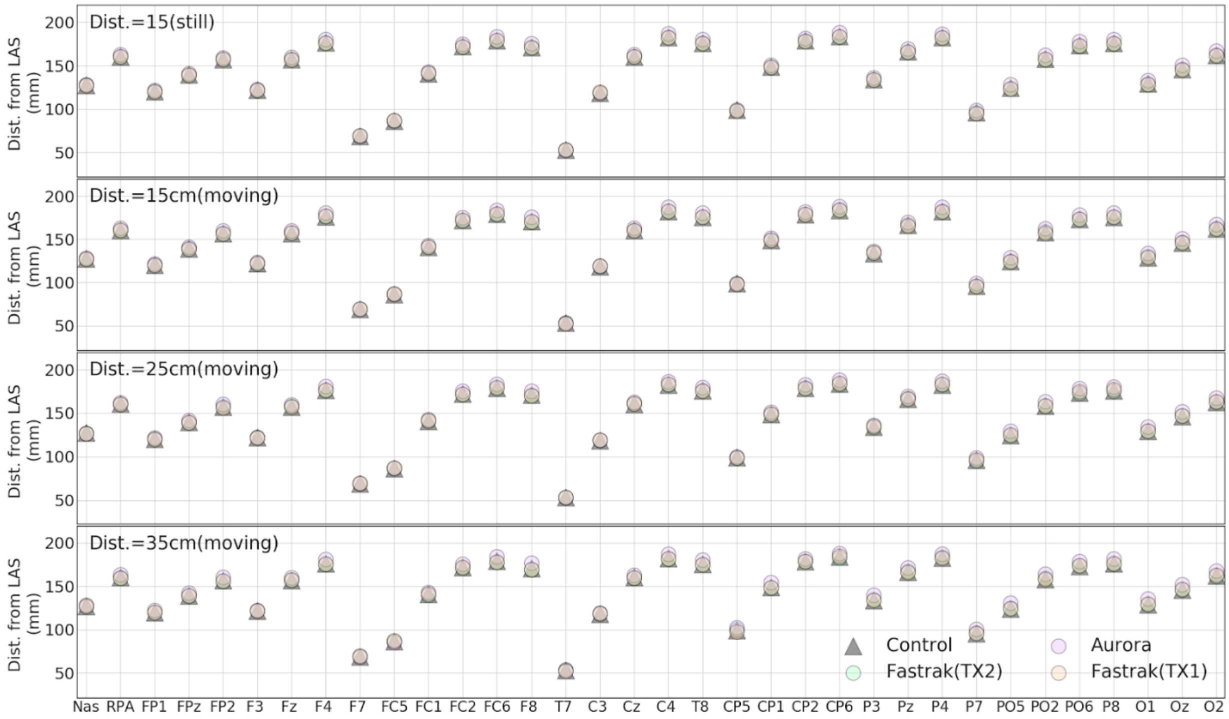


(B)

**Figure S5.** For 32-channel EEG cap A) Digitization error in different conditions; B) Distances between LPA and rest 34 locations (32 electrodes and 2 fiducials).

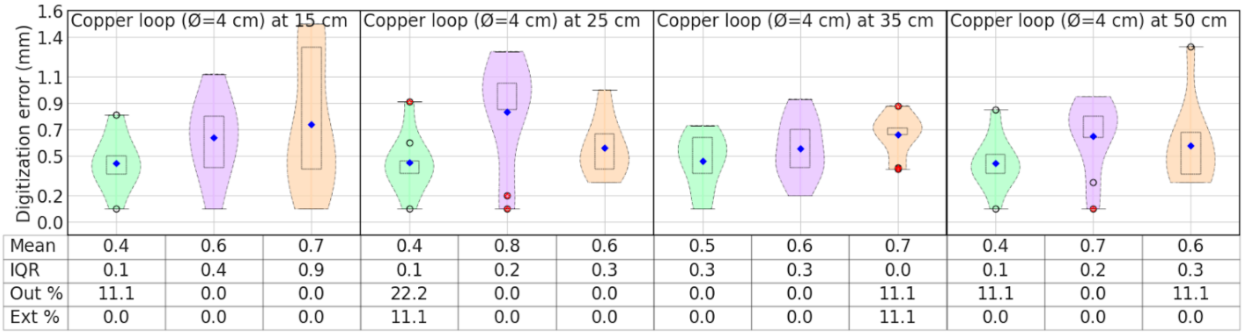


| | | | | | | | | | | | | |
|---|---|---|---|---|---|---|---|---|---|---|---|---|
| Mean | 0.4 | 0.6 | 0.7 | 0.4 | 0.8 | 0.6 | 0.5 | 0.6 | 0.7 | 0.4 | 0.7 | 0.6 |
| IQR | 0.1 | 0.4 | 0.9 | 0.1 | 0.2 | 0.3 | 0.3 | 0.3 | 0.0 | 0.1 | 0.2 | 0.3 |
| Out % | 11.1 | 0.0 | 0.0 | 22.2 | 0.0 | 0.0 | 0.0 | 0.0 | 11.1 | 11.1 | 0.0 | 11.1 |
| Ext % | 0.0 | 0.0 | 0.0 | 11.1 | 0.0 | 0.0 | 0.0 | 0.0 | 11.1 | 0.0 | 0.0 | 0.0 |

(A)

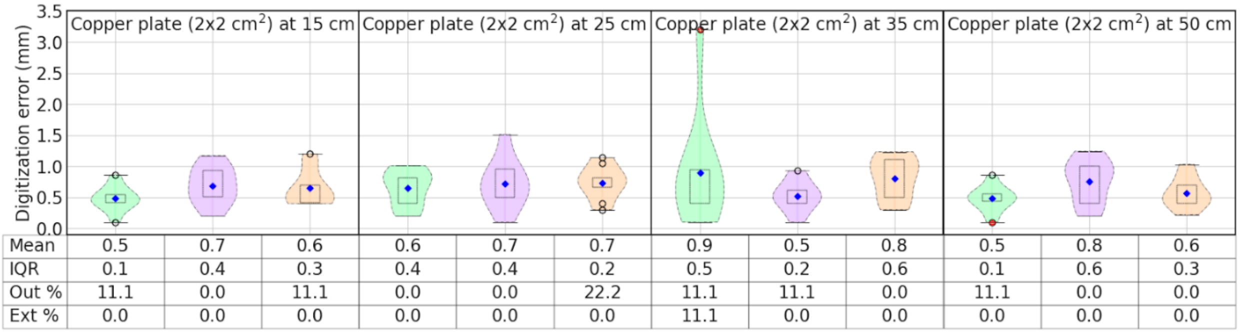


| | | | | | | | | | | | | |
|---|---|---|---|---|---|---|---|---|---|---|---|---|
| Mean | 0.5 | 0.7 | 0.6 | 0.6 | 0.7 | 0.7 | 0.9 | 0.5 | 0.8 | 0.5 | 0.8 | 0.6 |
| IQR | 0.1 | 0.4 | 0.3 | 0.4 | 0.4 | 0.2 | 0.5 | 0.2 | 0.6 | 0.1 | 0.6 | 0.3 |
| Out % | 11.1 | 0.0 | 11.1 | 0.0 | 0.0 | 22.2 | 11.1 | 11.1 | 0.0 | 11.1 | 0.0 | 0.0 |
| Ext % | 0.0 | 0.0 | 0.0 | 0.0 | 0.0 | 0.0 | 11.1 | 0.0 | 0.0 | 0.0 | 0.0 | 0.0 |

(B)

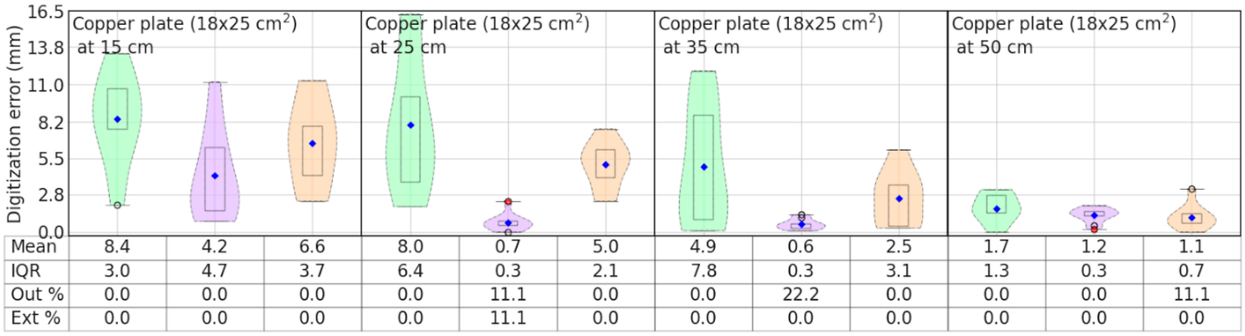


| | | | | | | | | | | | | |
|---|---|---|---|---|---|---|---|---|---|---|---|---|
| Mean | 8.4 | 4.2 | 6.6 | 8.0 | 0.7 | 5.0 | 4.9 | 0.6 | 2.5 | 1.7 | 1.2 | 1.1 |
| IQR | 3.0 | 4.7 | 3.7 | 6.4 | 0.3 | 2.1 | 7.8 | 0.3 | 3.1 | 1.3 | 0.3 | 0.7 |
| Out % | 0.0 | 0.0 | 0.0 | 0.0 | 11.1 | 0.0 | 0.0 | 22.2 | 0.0 | 0.0 | 0.0 | 11.1 |
| Ext % | 0.0 | 0.0 | 0.0 | 0.0 | 11.1 | 0.0 | 0.0 | 0.0 | 0.0 | 0.0 | 0.0 | 0.0 |

(C)

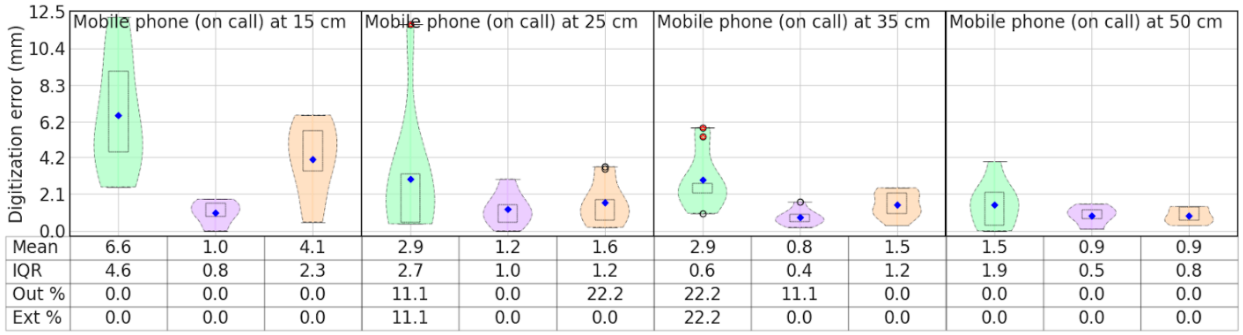


| | | | | | | | | | | | | |
|---|---|---|---|---|---|---|---|---|---|---|---|---|
| Mean | 6.6 | 1.0 | 4.1 | 2.9 | 1.2 | 1.6 | 2.9 | 0.8 | 1.5 | 1.5 | 0.9 | 0.9 |
| IQR | 4.6 | 0.8 | 2.3 | 2.7 | 1.0 | 1.2 | 0.6 | 0.4 | 1.2 | 1.9 | 0.5 | 0.8 |
| Out % | 0.0 | 0.0 | 0.0 | 11.1 | 0.0 | 22.2 | 22.2 | 11.1 | 0.0 | 0.0 | 0.0 | 0.0 |
| Ext % | 0.0 | 0.0 | 0.0 | 11.1 | 0.0 | 0.0 | 22.2 | 0.0 | 0.0 | 0.0 | 0.0 | 0.0 |

(D)

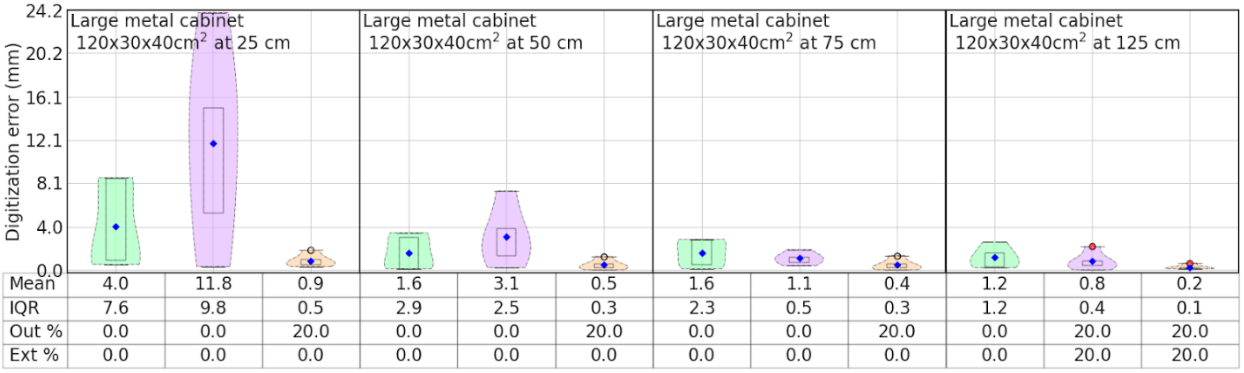


| | | | | | | | | | | | | |
|---|---|---|---|---|---|---|---|---|---|---|---|---|
| Mean | 4.0 | 11.8 | 0.9 | 1.6 | 3.1 | 0.5 | 1.6 | 1.1 | 0.4 | 1.2 | 0.8 | 0.2 |
| IQR | 7.6 | 9.8 | 0.5 | 2.9 | 2.5 | 0.3 | 2.3 | 0.5 | 0.3 | 1.2 | 0.4 | 0.1 |
| Out % | 0.0 | 0.0 | 20.0 | 0.0 | 0.0 | 20.0 | 0.0 | 0.0 | 20.0 | 0.0 | 20.0 | 20.0 |
| Ext % | 0.0 | 0.0 | 0.0 | 0.0 | 0.0 | 0.0 | 0.0 | 0.0 | 0.0 | 0.0 | 20.0 | 20.0 |

(E)

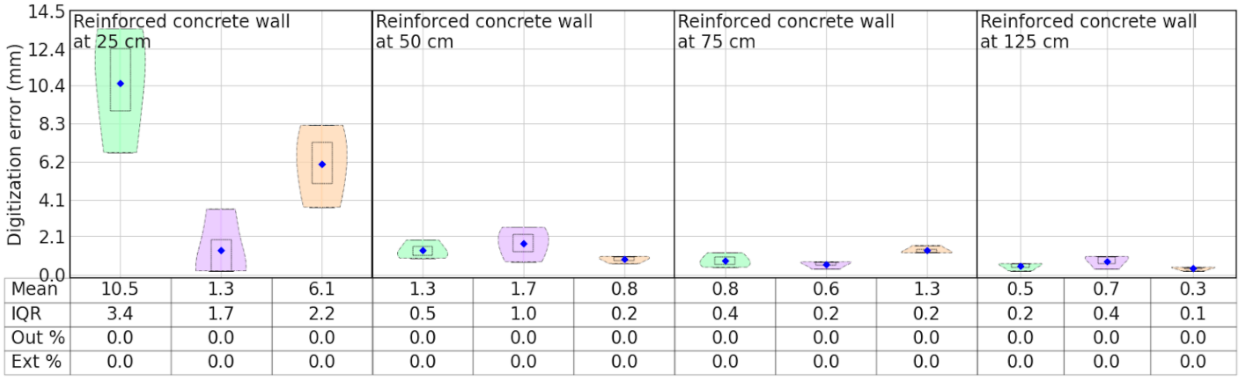


| | | | | | | | | | | | | |
|---|---|---|---|---|---|---|---|---|---|---|---|---|
| Mean | 10.5 | 1.3 | 6.1 | 1.3 | 1.7 | 0.8 | 0.8 | 0.6 | 1.3 | 0.5 | 0.7 | 0.3 |
| IQR | 3.4 | 1.7 | 2.2 | 0.5 | 1.0 | 0.2 | 0.4 | 0.2 | 0.2 | 0.2 | 0.4 | 0.1 |
| Out % | 0.0 | 0.0 | 0.0 | 0.0 | 0.0 | 0.0 | 0.0 | 0.0 | 0.0 | 0.0 | 0.0 | 0.0 |
| Ext % | 0.0 | 0.0 | 0.0 | 0.0 | 0.0 | 0.0 | 0.0 | 0.0 | 0.0 | 0.0 | 0.0 | 0.0 |

(F)

**Figure S6.** Digitization accuracy of the three systems when (A) a copper loop (Ø = 4 cm), (B) a small copper plate (2 cm x 2 cm), (C) a large copper plate (18 cm x 25 cm), (D) an on-call mobile phone, E) a large (1.2 m x 0.3 m x 0.4 m) metal cabinet, F) a steel reinforced concrete (RCC) wall, was at varying distances (mentioned in plots titles) from the transmitter.

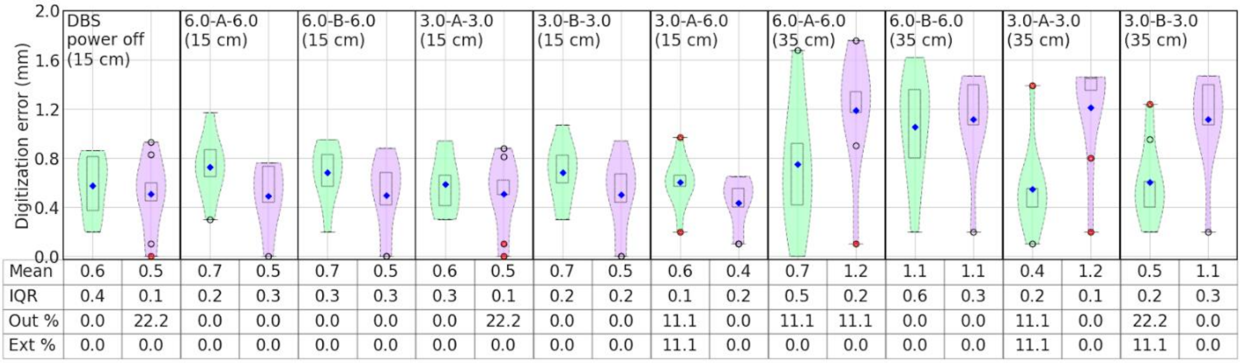


| | | | | | | | | | | | | | | | | | | | | |
|---|---|---|---|---|---|---|---|---|---|---|---|---|---|---|---|---|---|---|---|---|
| Mean | 0.6 | 0.5 | 0.7 | 0.5 | 0.7 | 0.5 | 0.6 | 0.5 | 0.7 | 0.5 | 0.6 | 0.4 | 0.7 | 1.2 | 1.1 | 1.1 | 0.4 | 1.2 | 0.5 | 1.1 |
| IQR | 0.4 | 0.1 | 0.2 | 0.3 | 0.3 | 0.3 | 0.3 | 0.1 | 0.2 | 0.2 | 0.1 | 0.2 | 0.5 | 0.2 | 0.6 | 0.3 | 0.2 | 0.1 | 0.2 | 0.3 |
| Out % | 0.0 | 22.2 | 0.0 | 0.0 | 0.0 | 0.0 | 0.0 | 22.2 | 0.0 | 0.0 | 11.1 | 0.0 | 11.1 | 11.1 | 0.0 | 0.0 | 11.1 | 0.0 | 22.2 | 0.0 |
| Ext % | 0.0 | 0.0 | 0.0 | 0.0 | 0.0 | 0.0 | 0.0 | 0.0 | 0.0 | 0.0 | 11.1 | 0.0 | 0.0 | 0.0 | 0.0 | 0.0 | 11.1 | 0.0 | 11.1 | 0.0 |

**Figure S7.** Digitization accuracy of the Fastrak TX2 and Aurora systems in the presence of an active DBS. The therapy setting and distance of the test model from the transmitter are mentioned in the plot legend.
